# Artificial Intelligence for COVID-19 Detection in Medical Imaging—Diagnostic Measures and Wasting—A Systematic Umbrella Review

**DOI:** 10.3390/jcm11072054

**Published:** 2022-04-06

**Authors:** Paweł Jemioło, Dawid Storman, Patryk Orzechowski

**Affiliations:** 1 AGH University of Science and Technology, Faculty of Electrical Engineering, Automatics, Computer Science and Biomedical Engineering, al. A. Mickiewicza 30, 30-059 Krakow, Poland; patryk.orzechowski@gmail.com; 2 Chair of Epidemiology and Preventive Medicine, Department of Hygiene and Dietetics, Jagiellonian University Medical College, ul. M. Kopernika 7, 31-034 Krakow, Poland; dawid.storman@doctoral.uj.edu.pl; 3 Institute for Biomedical Informatics, University of Pennsylvania, 3700 Hamilton Walk, Philadelphia, PA 19104, USA

**Keywords:** COVID-19, diagnosis, artificial intelligence, medical imaging, systematic umbrella review, methodological credibility

## Abstract

The COVID-19 pandemic has sparked a barrage of primary research and reviews. We investigated the publishing process, time and resource wasting, and assessed the methodological quality of the reviews on artificial intelligence techniques to diagnose COVID-19 in medical images. We searched nine databases from inception until 1 September 2020. Two independent reviewers did all steps of identification, extraction, and methodological credibility assessment of records. Out of 725 records, 22 reviews analysing 165 primary studies met the inclusion criteria. This review covers 174,277 participants in total, including 19,170 diagnosed with COVID-19. The methodological credibility of all eligible studies was rated as *critically low*: 95% of papers had significant flaws in reporting quality. On average, 7.24 (range: 0–45) new papers were included in each subsequent review, and 14% of studies did not include any new paper into consideration. Almost three-quarters of the studies included less than 10% of available studies. More than half of the reviews did not comment on the previously published reviews at all. Much wasting time and resources could be avoided if referring to previous reviews and following methodological guidelines. Such information chaos is alarming. It is high time to draw conclusions from what we experienced and prepare for future pandemics.

## 1. Introduction

In early December, 2019, a new coronavirus epidemic was identified in Wuhan [1]. Coronavirus disease 2019 (COVID-19) is a viral infection spread by direct contact with people experiencing the illness (from droplets generated by sneezing and coughing) or indirectly [2]. It is caused by Severe Acute Respiratory Syndrome Coronavirus 2 (SARS-CoV-2). As of 23 February 2022, over 420 million people have been diagnosed with COVID-19, with nearly 5.89 million associated deaths [3]. The consecutive waves of COVID-19 affected many societies, as well as scientific foundations and organisations [4,5,6,7]. On 30 January 2020, the World Health Organisation (WHO) issued a public health emergency of international concern (PHEIC) associated with COVID-19 and declared the state of a pandemic on 11 March 2020 [8].

Disease manifestation is variable, with some infected people remaining asymptomatic (even up to 57% [9]) and others suffering from mild (including fever, cough, and aches) to severe (involving lethargy with dyspnoea and increased respiratory rate) and critical manifestations (requiring mechanical ventilation). It may lead to serious neurological, musculoskeletal, or cerebrovascular disorders or may even progress to a life-threatening respiratory syndrome in some patients [10,11].

Moreover, in 80% of patients, COVID-19 may leave one or more long-lasting symptoms, with fatigue, headaches, attentional difficulties, anosmia, and memory loss manifesting the most frequently [12]. Wide-ranging longer-term morbidity has also been described in the absence of severe initial illnesses [13].

The essence of stopping the significant increase in morbidity is, in addition to treatment, quick diagnostics. The identification of those infected allows for better management of the pandemic (e.g., isolation, quarantine, hospital admission or admission to the intensive care unit) [14]. Understanding the accuracy of tests and diagnostic features seems essential to develop effective screening and management methods [15].

As the pandemic unfolded, many ways have been found to diagnose COVID-19. The primary method for diagnosing COVID-19 is Nucleic Acid Amplification Tests (NAATs). It utilises respiratory tract samples (mainly from the nasopharynx or oropharynx). However, some guidelines recommend nasal swabs [16], and some evidence suggests lower respiratory samples, such as sputum, may have higher sensitivity [17].

From the pandemic onset, chest radiography (X-ray) has been a helpful tool for COVID-19 diagnosis [18]. Nevertheless, even routine chest radiography does not confirm that the patient has COVID-19, especially early on [19], so diagnosing based on an X-ray is challenging. On the contrary, a computed tomography (CT) has been able to discover COVID-19 abnormalities with sensitivity exceeding 97% [20]. However, it was reported to have only 25% to 83% specificity for symptomatic patients [21]. Some evidence suggests it helps to detect COVID-19 earlier than manifested by the positive reverse transcription-polymerase chain reaction (RT-PCR) test [22,23]. Additionally, from the beginning of the pandemic, COVID-19 diagnosis based on ultrasound imaging proved to be of sensitivity and accuracy, which is similar to differentiating with CTs [24].

With the rising role of medical imaging as a diagnostic tool for COVID-19, a question arose if and to which extent automated tools could be included in clinical diagnosis. Up to this day, artificial intelligence (AI), or more specifically, deep learning (DL), have started to play an increasingly vital role in medicine [25]. AI can be employed in the first step of diagnosis, or the results it produces may be used to confirm hypotheses generated by clinicians. In some recent studies and clinical trials, AI has been demonstrated to match or even exceed the performance of expert radiologists, which could potentially offer expedited and less expensive diagnostics [26,27,28,29,30,31]. A study and meta-analysis by [32] with 31,587 identified and 82 included studies shows DL is even capable of slightly outperforming health care professionals in detecting diseases from medical images with a pooled sensitivity of 87% (vs. 86%) and a pooled specificity of 93% (vs. 91%), respectively.

Since the emergence of the COVID-19 pandemic, around 237,000 related papers (and growing) have been published [33,34]. The urgency of reporting novel findings and high pressure to publish COVID-19-related research quickly has been reported to lead to exceptions to high standards of quality [35,36], an increase in overlap [37], lowering methodological credibility of some of the articles [38], or even accepting papers with numerous analytical errors [39].

Almost two years after the pandemic onset, it is the right time to start drawing conclusions [40]. We should also pay attention to the mistakes we have committed and avoid them in the face of the upcoming threats. The current situation is an opportunity to learn lessons on dealing with crises.

This systematic umbrella review aims to screen reviews on AI techniques to diagnose COVID-19 in patients of any age and sex (both hospitalised and ambulatory) using medical images and assess their methodological quality. Additionally, our goal was to evaluate the research publishing process and the degree of overlap to assess the legitimacy of creating new works in the unfolding pandemic.

## 2. Materials and Methods

### 2.1. Data Sources and Searches

In order to determine whether there are any eligible papers, we conducted a pre-search in the middle of August 2020 via Google Scholar by browsing. Next, we searched seven article databases (MEDLINE, EMBASE, Web of Science, Scopus, dblp, Cochrane Library, IEEE Xplore) and two preprint databases (arXiv, OSF Preprints) from inception to 1 September 2020 using predefined search strategies. In developing the search strategy for MEDLINE, we combined the Medical Subject Headings (MeSH) and full-text words. In Text S1, we present the used strategies. No date or language restrictions were adopted. Additionally, we searched the references of included studies for eligible records.

### 2.2. Study Selection

We focused on any review (systematic or not) that includes primary studies utilising AI methods with medical imaging results to diagnose COVID-19. We were particularly interested in the performance of such classification systems, e.g., accuracy, sensitivity, specificity. Based on available guidelines [16], we excluded these primary studies that used reference standards other than assay types (NAATs, antigen tests, and antibody tests) from nasopharyngeal or oropharyngeal swab samples, nasal aspirate, nasal wash or saliva, sputum or tracheal aspirate, or bronchoalveolar lavage (BAL) [41].

Additionally, due to overlapping and double referencing of the post-conference articles (particular chapters), we excluded entire proceedings and post-conference books as they contain little information about the topics (presented in chapters) *per se*. However, we did not exclude reviews (chapters) as they were still present in our search.

The protocol of this review was published [42] and registered [43] on the OSF platform.

Using Endnote X8 (Clarivate Analytics ^®^) and Rayyan [44], we checked identified references for duplicates. P.J., D.S., and P.O. independently screened the remaining references using the latter application, and subsequently, independently assessed the full texts for meeting the inclusion criteria.

To improve the understanding of the criteria among the reviewers, we carried out pilot exercises before the screening of titles and abstracts and full texts assessment. We achieved consensus via discussion if any conflicts occurred.

### 2.3. Definitions

We defined the terms used in our eligibility criteria below. *Review* refers to a paper identified by authors as a review or a survey. *AI* refers to computer programs that can perform tasks as intelligent beings [45]. *COVID-19* refers to a disease caused by the SARS-CoV-2 virus [46]. *Imaging* refers to individuals’ medical imaging results (e.g., CT scans, X-rays, ultrasound images) [47,48].

*Diagnosis* refers to the identification of an illness (here: COVID-19) [49]. *Performance metrics* refers to evaluating machine learning algorithms. These measures are utilised to juxtapose observed data (actual labels) with the predictions of the model [50].

### 2.4. Data Extraction and Quality Assessment

Before the extraction phase, we checked included preprints for peer-reviewed versions and included them, if available. We predefined an extraction form, and P.J. and D.S. collected all necessary data independently. We gathered information about authors, funding, population, models, outcomes—AI diagnostic metrics, and additional analyses.

We also extracted bibliometric data about publishing dates (availability), sending to the editors (first and last version), and acceptance in a journal or a conference of included reviews. Moreover, we checked the availability dates for primary studies.

To provide a common understanding of the criteria, we performed calibration exercises before data extraction and credibility assessment. When the conflict occurred, we discussed the final version.

P.J. and D.S. conducted quality evaluations independently. We assessed the methodological credibility using AMSTAR 2 [51] with critical items (2, 4, 7, 9, 11, 13, and 15), indicated as such by AMSTAR 2 authors, and not yet validated extended version of QASR [52].

The general quality across the study was evaluated as *critically low* when more than one item in a critical domain was considered a flaw [51].

In this paper, we concentrate only on the results of applying AMSTAR 2 (as it is suggested for evaluating systematic reviews [53]), while a full assessment of both instruments will be included in the next methodologically focused article.

Additionally, we assessed the quality of reporting in included studies using the Preferred Reporting Items for Systematic Reviews and Meta-analyses for Diagnostic Test Accuracy (PRISMA-DTA) checklist [54]. We rated each module on the 3-item scale: 0 (*no* with no compliance), 0.5 (*partial yes* with fragmentary compliance), 1 (*yes* with total compliance). Next, the results were summed, and the overall score was then assigned.

Based on the method of Li et al. [55] and taking into account two more items in the DTA extension [54] (comparing to the original instrument [56]), we differentiated the quality of reporting as follows:

- *Major flaws* when the final score was ≤17.0,
- *Minor flaws* when the final score was ≥17.5 and ≤23.0,
- *Minimal flaws* when the final score was ≥23.5.

In the case of reviews without meta-analysis, we lowered the cut-offs by 1 point following PRISMA-DTA [54].

### 2.5. Data Synthesis and Analysis

In this umbrella review, we focus on the descriptive summary of included papers regarding the quality and reporting on the most significant characteristics, such as population, models, interpretability, and outcomes.

We did not synthesise the results quantitatively because of the quality of included reviews, the agreement between them, and the percentage of non-reported data (data we intended to extract, e.g., accuracy of diagnostic methods or AI model type, see Section 3). Therefore, we do not present a subgroup analysis and investigation of heterogeneity, sensitivity, and publication bias analyses.

As for the in-depth characteristics, all the primary studies were divided into two groups: included in one review only and included in at least one review. The studies included in more than one review were analysed in 2 ways (A and B) considering *not reported* data. In analysis A, whenever *not reported* data from one review occurred together with a specific value from the other paper, we considered it *non-overlapping* and excluded it. In analysis B, we ignored *not reported* data and included the specific value. After the exclusion of non-overlapping data for continuous variables, we calculated the statistics, namely means with ranges.

For diagnostics metrics, we prepared the scatter plots. We considered data regardless of non-overlapping. Whenever disagreements between reviews occurred (in specific primary studies), we averaged the values. In case of lack of data (*not reported*), we provided two charts with modal imputation and without it.

From the above analyses, we excluded those primary studies, which included more than one DL model.

We analysed how extensive was the search performed by the authors of the reviews, i.e., percentage of the identified primary studies available up to the selected date. In the first case, we considered the reference date, by which we mean the day that the review was either received, accepted by the editors, or published. In the second analysis, we relaxed this condition to the date the last cited paper included in the review was available.

Investigating the citations between the reviews, we considered two different scenarios: citing only published reviews and citing both published and preprint versions.

We assessed inter-review agreement only if at least two different reviews included the same paper (and only one DL model). We determined the inter-review agreement as a percentage of overlapping values within all extracted data (text and non-text) and subgroups of characteristics (text and non-text) and outcomes. The text variables considered the dataset used, architecture, and post-processing. The inter-review agreement was assessed in 2 ways (analyses A and modified B—exclude a pair, instead of ignoring it).

All analyses were conducted using Python 3.7.10 (including libraries: Matplotlib 3.2.2, Seaborn 0.11.1, Pandas 1.1.5 and NumPy 1.19.5).

## 3. Results

### 3.1. Included Studies

**Figure 1:**
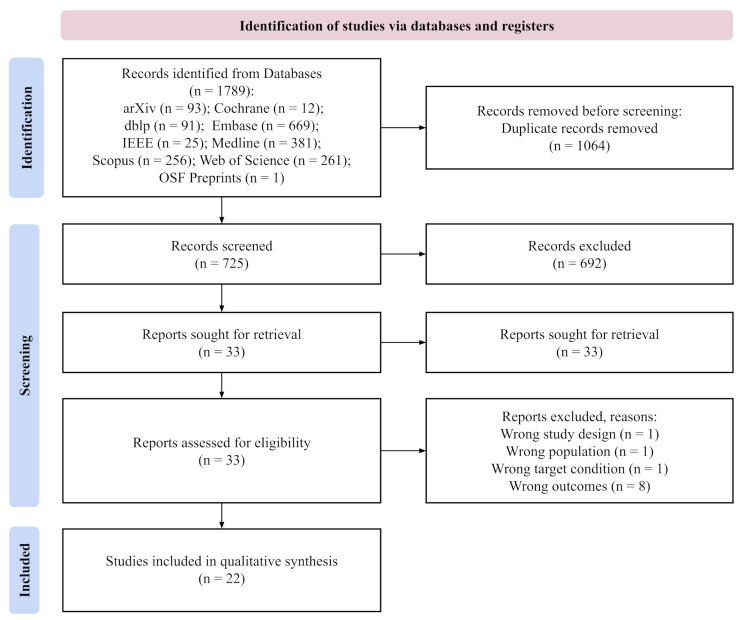
PRISMA flow chart.

After removing duplicates, we screened 725 studies, of which 33 were read in the form of full texts. In total, we included 22 reviews [57,58,59,60,61,62,63,64,65,66,67,68,69,70,71,72,73,74,75,76,77,78] for qualitative synthesis. We followed PRISMA guidelines [56]. The full study flow is presented in Figure 1.

The included and excluded studies (with reasons) are presented in Tables S1 and S2, respectively. The detailed characteristics of included reviews are shown in Table 1, while its extended version is ensured in Table S3.

We present the in-depth characteristics of primary studies in Table S4. Tables S5 and S6 focus on the non-overlapping between reviews and non-reporting in terms of specific extracted variables. Figures S1–S6 introduces visualised diagnostic metrics.

None of the reviews provided information about ethnicity, smoking, and comorbidities. Only one review [68] reported on age and gender proportion as well as the study design of discussed primary papers. None of the studies conducted a meta-analysis, but one summarised the results with the use of averages [71].

The analysed reviews described 165 primary papers (on average: eight primary studies per review, range: 1–11). Of these, 138 of them were included in at least one review, of which 73 were included only in one. Only 27 of the primary studies considered more than one DL model for diagnosis.

### 3.2. Quality of Included Studies

**Figure 2:**
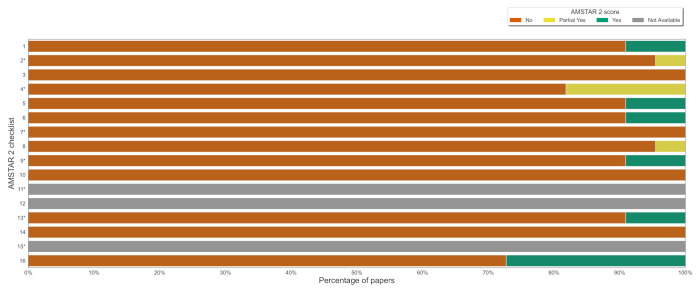
Quality graph: our judgements on each AMSTAR 2 item presented as the percentage of all the included studies; * denotes critical domains.

The general quality of all included studies is *critically low* (see Figure 2). Six reviews [62,63,64,66,68,77] provided full information about sources of funding and conflict of interest. It was the most satisfied item. None of the studies provided a list of excluded papers, explanation of eligible study design, and sources of funding in included studies.

A heatmap with the authors’ judgements regarding AMSTAR 2 items can be found in Figure S7. In Figure S8, we also included results per specific review.

**Figure 3:**
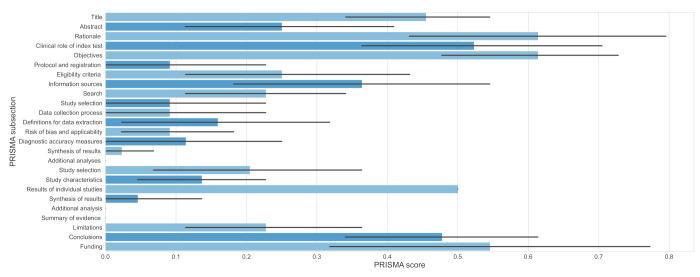
Quality of reporting graph: our judgements about each PRISMA-DTA item presented as averages (with 95% confidence intervals—black lines) across all included studies. Different shades of blue are used just to improve the chart’s clarity.

In terms of reporting, *major flaws* are present among 21 of the included papers. Only one review [68] contains *minor flaws* (see Figure 3 and Figures S9 and S10). The most affected domains were those concerning additional analyses both in terms of methods and results (all reviews). Similarly, a summary of evidence was not reported in any review.

On the contrary, 12 of the included papers [57,59,62,63,64,66,68,69,70,71,77,78] reported fully on funding. Additionally, 11 reviews [57,58,60,65,66,69,70,72,73,76,77] and eight reviews [57,59,60,65,66,67,71,77] described the rationale and objectives in the introduction adequately. These were the most satisfied domains.

The mean overall score of reporting quality across the included reviews equals 6.23 (1.5–17.5). Across all items and studies, the most frequent score was 0 (*no*) with 67%; 19% of the time, we assessed the items as 0.5 (*partially yes*). A heatmap with all authors’ judgements regarding PRISMA-DTA items can be found in Figure S9. In Figure S10, we also included summarised results per specific review.

### 3.3. Resources and Time Wasting Analyses

**Figure 4:**
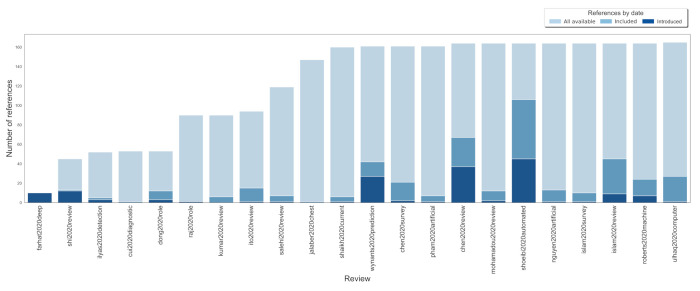
The cumulative chart of included, available (by the date), and introduced primary papers among discussed reviews.

The included studies were published or available online without peer reviewing from 11 April 2020 to 12 October 2020 (see Figure S11). In Figure 4, we presented a cumulative chart of all 165 primary studies included in the discussed reviews.

The number of included interesting studies (related to our research question) in selected reviews ranged from 1 [62,63,64] to 106 [73]. Moreover, we present the percentage of articles introduced by (first appear in) a particular review. Figure S11 additionally depicts the appearance of included reviews and interesting primary studies over time.

Half (50%) of all (165) primary studies (the half-saturation constant) were included at least once before the end of July 2020. However, the same number of papers was available for inclusion three months earlier.

Next, we investigated the extent to which review authors performed the search. Regarding the reference date (see Figure S11), the mean percentage of the primary studies covered was 14% (1–64). When relaxed, the mean percentage of covered studies increased to 24% (1–65). More details about the search are presented in Table S7.

Out of all the studies, 14% did not include any new paper into consideration. The mean primary studies that were introduced by a particular review was 7.24 (0–45).

Analysing published versions of reviews only, the cross-citing equals 0.81 (0–4). Including also preprints, 1.1 (0–7) published papers or preprints were quoted by the authors of the subsequent reviews. Notably, 12 (55%) of reviews did not refer to any previously available ones at all.

Figures S12–S21 present results regarding the agreement between reviews (*pairs of reviews*) in the reporting of characteristics and outcomes.

## 4. Discussion

Generally, we report that the quality of the included reviews was *critically low*. Similar findings were found by Jung et al. [79], who observed lowered methodological credibility in 686 of 14,787 screened COVID-19 papers. Analyses of reviews on COVID-19 by Yu et al. [80] and Al-Ryalat et al. [81] also showed their unsatisfactory credibility. It adds on top of the generally low quality of reporting of DL performance from medical images, with a high risk of bias present in 58 out of 81 of the existing studies (72%) [82].

Poor quality is not related only to COVID-19 and AI. Still, it occurs in many fields such as bariatric surgery with up to 99% *critically low* articles [83], psychology with 95% of papers [84], or methodology where 53 out of 63 publications were of *critically low* quality [85].

What is more, we also noticed major flaws in reporting. Nagendran et al. [82] observed the same, but they used the original PRISMA instrument. In our research, three PRISMA-DTA domains were fully violated by all reviews. Although the authors focused on diagnostics, they poorly reported on accuracy measures and explicit description of the extraction process. None of the included studies performed a meta-analysis, similar to what Adadi et al. [86] found in their study.

The low credibility of evidence and flawed reporting (e.g., population characteristics) can be associated with a lack of knowledge of reporting standards and clinical practice or misunderstandings regarding AI methods and additional analyses.

We also observed multiple disagreements between the included reviews. However, excluding them from synthesis is associated with a vast information loss, e.g., the number of participants. Inconsistencies were also noticed in the reporting of DL architecture. For instance, the following names were used across multiple studies: *ResNet-18*, *ResNet18*, *resnet-18*, *18-layer ResNet*. In some of the papers, the architecture was not reported at all. It made it challenging to group models into similar subsets. Some discrepancy was also observed in extracting the measurements of AI models performance, e.g., diagnostic effectiveness metrics. Such negligence may lead to further replicating the errors by subsequent studies and should be corrected before releasing the paper or soon after in an updated version or in an associated erratum.

Many of the reviews included in this paper did not strengthen the evidence on using AI in diagnosing COVID-19 from medical imaging. Those works have not identified and correctly cited pre-existing primary papers, which is deemed the essence of any research. Some of the potential explanations are that multiple similar studies might have been initiated around the same time, and prolonged review times impacted their content. Alternatively, the research objectives of some articles were broad enough to preclude a deeper analysis of the use of artificial intelligence in medical imaging.

Wasting may also (or in particular) be observed on the primary studies level. Failure to consider previous results may lead to publishing new papers describing models similar to those presented by other researchers. Shockingly, these newer DL architectures rely sometimes on fewer participants or COVID-19 cases, so they probably reflect reality less adequately. As researchers suggest, the amount of waste and poor biomedical research quality is staggering [35,87]. Papers that do not bring any additional evidence to the field can be considered redundant [88,89,90,91].

Proper reporting of deep learning performance from primary studies is challenging. Naudé [92] has pointed out some of the significant concerns regarding the adoption of AI in COVID-19 research, including data availability and its quality. Still, many studies do not ensure that the utilised code is open-source, which highly limits the reproducibility of their findings [93,94]. Therefore, we suggest sharing it so that you can react faster and more effectively from the perspective of the upcoming, similar breakdowns.

On average, when not considering credibility issues, the diagnostic metrics of described models exceed the human’s ability to diagnose COVID-19 from medical imaging [32]. Sadly, no evidence of such an advantage could be transferred to any implications for practice because of not following reporting and quality instruments. It resembles a situation when a long jumper did not break the world record just because they stepped on the foul line.

### Study Strengths and Limitations

Our umbrella review has the following strengths. First, the search strategy was comprehensive. It is based on adequate inclusion criteria related to the research question and spanned across a wide selection of existing data sources: papers and preprints. This selection was further expanded by searching the references of included papers to identify additional works. It is noteworthy that the searches were not limited in terms of format or language (we imposed no restrictions). The process of our review was rigorous as the study was preceded by the publication of protocol. We used the most up-to-date and applicable instruments to assess the credibility and quality of reporting—AMSTAR 2 and PRISMA with extension for DTA, respectively.

Nevertheless, these two have been designed for reviews in medicine and health sciences, where the formulation of the research question is structured, the methodology is validated, and the quantitative synthesis of results is popular.

It must be noted, though, that the vast majority of the included studies focused on a broader context than purely diagnosing COVID-19 from medical images.

In this study, we have also investigated wasting among the reviews. We based on the date of publishing of the last included primary study in a specific review. By doing so, we aimed at assessing the depth of the search performed by the authors. We assumed that if the authors included a given study, they should have had the required knowledge about all the papers available before it was released. This approach relaxes the strict requirement to include all the studies that appeared before the review was published and seems to measure the quality of the search more objectively.

The level of agreement between reviews differs remarkably depending on the extracted variable, so without comparing with the primary studies, we cannot be fully convinced of the data correctness reported in the reviews. In the assessment of the inter-review agreement, we considered only these reviews that included the same paper (and described one DL model).

## 5. Conclusions

The COVID-19 research is quickly moving forward. Each day hundreds of new papers are published [95,96,97]. As AI starts to play an increasingly important role in clinical practice [98,99], it is crucial to evaluate its performance correctly.

In this paper, we synthesised and assessed the quality of the 22 reviews that mention using AI on COVID-19 medical images. We reviewed them and critically assessed their reporting and credibility using well-established instruments such as PRISMA-DTA [54] and AMSTAR 2 [51].

We explored the beginning of the pandemic when much uncertainty and confusion existed in the world of science. It seems that the number of articles and the pace of their publishing during the future global outbreaks might be even faster. Thus, it is essential to draw the appropriate conclusions now and treat this *briefing* as an opportunity to optimise work and avoid wasting in publishing.

In order to accomplish this, we urge the authors of the reviews to use PRISMA [56] and AMSTAR 2 [51] and the authors of primary studies to follow appropriate tools [100,101].

It is high time to adopt best practices, improve the research quality, and apply higher scrutiny in filtering out non-constructive contributions.

## Figures and Tables

**Table 1:** Detailed characteristics of included reviews.

Variable	Number (Percentage)	Mean (Range)^2^
Number of reviews with the authors from a specific country		
United States of America	8 (18%)	NA
Australia	4 (9%)	NA
China	4 (9%)	NA
India	4 (9%)	NA
United Kingdom	3 (7%)	NA
Other	22 (49%)	NA
Total number of authors of the reviews	171	8 (1-43)
Type of publication		
Journal article (mean IF^1^: 4.14; range: 0–30.31)	13 (59%)	NA
*IEEE Access*	2 (9%)	NA
*IEEE Reviews in Biomedical Engineering*	2 (9%)	NA
*Diagnostic and Interventional Imaging*	2 (9%)	NA
*Diabetes & Metabolic Syndrome*: *Clinical Research & Reviews*	1 (5%)	NA
*Applied Intelligence*	1 (5%)	NA
*British Medical Journal*	1 (5%)	NA
*Biosensors and Bioelectronics*	1 (5%)	NA
*Machine Vision and Applications*	1 (5%)	NA
*Current Problems in Diagnostic Radiology*	1 (5%)	NA
*Journal of the Indian Medical Association*	1 (5%)	NA
Preprint article	8 (36%)	NA
Conference article	1 (5%)	NA
Was the review specified as systematic by the authors?		
No	20 (91%)	NA
Yes	2 (9%)	NA
Number of reviews that searched a given data source	50	5 (3-7)
arXiv	8 (36%)	NA
medRxiv	6 (27%)	NA
Pubmed/Medline	6 (27%)	NA
Google Scholar	6 (27%)	NA
bioRxiv	5 (23%)	NA
IEEE Xplore	3 (14%)	NA
Science Direct	3 (14%)	NA
ACM digital library	2 (9%)	NA
Springer	2 (9%)	NA
MICCAI conference	1 (5%)	NA
IPMI conference	1 (5%)	NA
Embase	1 (5%)	NA
Web of Science	1 (5%)	NA
Elsevier	1 (5%)	NA
Nature	1 (5%)	NA
Number of studies		
Reported by review authors as included	358	51 (20–107)
Applicable for this review question (total)	451	21 (1–106)
Applicable for this review question (unique only)	165	7.5 (0–11)

## Data Availability

Code used in this research is available on GitHub: https://github.com/pawljmlo/covid-ur-wasting (accessed on 25 March 2022). The data presented in this study are available on request from the corresponding author.

## Supplementary Materials

The following supporting information can be downloaded at: https://www.mdpi.com/article/10.3390/jcm11072054/s1, **Table S1**: Included studies with dates: first received, last received, accepted and published; **Table S2**: Exluded studies with reasons; **Table S3**: Full characteristics of included reviews [102,103,104,105,106,107,108,109,110,111,112]; **Table S4**: In-depth characteristics of primary studies; **Table S5**: Non-overlapping of in-depth characteristics variables; **Table S6**: Non-reporting of in-depth characteristics variables; **Figure S1**: CT-based COVID-19 diagnosis, without imputation; **Figure S2**: CT-based COVID-19 diagnosis, with modal imputation; **Figure S3**: X-Ray-based COVID-19 diagnosis, without imputation; **Figure S4**: X-Ray-based COVID-19 diagnosis, with modal imputation; **Figure S5**: COVID-19 diagnosis with full patients number data provided, without imputation; **Figure S6**: COVID-19 diagnosis with any patients number data provided, with modal imputation; **Figure S7**: Review authors’ judgements about each AMSTAR 2 item across all included studies; * denotes critical items; **Figure S8**: AMSTAR 2 score in each included review; **Figure S9**: Review authors’ judgements about each PRISMA-DTA item across all included studies; **Figure S10**: PRISMA-DTA score in each included review; **Figure S11**: Appearance of included reviews (vertical lines, reference date, see Table S1 and interesting primary studies (dots); **Table S7**: Time and resource wasting statistics; **Figure S12**: Level of agreement based on overlapping in extracted data (characteristics only, without text data) between included reviews; analysis A; **Figure S13**: Level of agreement based on overlapping in extracted data (characteristics only) between included reviews; analysis A; **Figure S14**: Level of agreement based on overlapping in extracted data (outcomes only) between included reviews; analysis A; **Figure S15**: Level of agreement based on overlapping in extracted data (all variables, without text data) between included reviews; analysis A; **Figure S16**: Level of agreement based on overlapping in extracted data (all variables) between included reviews; analysis A; **Figure S17**: Level of agreement based on overlapping in extracted data (characteristics only, without text data) between included reviews; analysis B; **Figure S18**: Level of agreement based on overlapping in extracted data (characteristics only) between included reviews; analysis B; **Figure S19**: Level of agreement based on overlapping in extracted data (outcomes only) between included reviews; analysis B; **Figure S20**: Level of agreement based on overlapping in extracted data (all variables, without text data) between included reviews; analysis B; **Figure S21**: Level of agreement based on overlapping in extracted data (all variables) between included reviews; analysis B; **Text S1**: Search Strategies; PRISMA 2020 Checklist.

## Abbreviations

The following abbreviations are used in this manuscript:

AI	artificial intelligence
BAL	bronchoalveolar lavage
COVID-19	Coronavirus disease 2019
CT	computed tomography
DL	deep learning
NAATs	Nucleic Acid Amplification Tests
PHEIC	public health emergency of international concern
X-ray	radiography
RT-PCR	reverse transcription-polymerase chain reaction
SARS-CoV-2	Severe Acute Respiratory Syndrome Coronavirus 2
WHO	World Health Organisation
