# Supplementary material for: Artificial Intelligence for COVID-19 Detection in Medical Imaging—Diagnostic Measures and Wasting—A Systematic Umbrella Review
Source: J Clin Med. 2022 Apr 6;11(7):2054. doi: 10.3390/jcm11072054 (PMC9000039; doi:10.3390/jcm11072054)
Supplement: Supplementary file 1 [file jcm-11-02054-s001.zip › jcm-1631539-Supplementary.pdf]

Supplementary Material for:

# Artificial Intelligence for COVID-19 Detection in Medical Imaging—Diagnostic Measures and Wasting—A Systematic Umbrella Review

Paweł Jemioło, Dawid Storman, Patryk Orzechowski
E-mail: pawljmlo@agh.edu.pl

**Table S1.** Included studies with dates: first received, last received, accepted and published.

| ID | Reference date | First received | Last received | Accepted | Published / Available online |
|---|---|---|---|---|---|
| [57] | **11.04** | 06.04 | **11.04** | 11.04 | 16.04 |
| [58] | **12.04** | 11.04 | **12.04** | 11.04 | 11.04 |
| [59] | **20.04** | 12.04 | **20.04** | 22.04 | 27.04 |
| [60] | **01.05** | 27.04 | **01.05** | 03.05 | 20.05 |
| [61] | **03.05** | 25.04 | **03.05** | 04.05 | 07.05 |
| [62] | **15.05** | 25.04 | Not Reported | **15.05** | Not Reported |
| [63] | **27.05** | 12.04 | **27.05** | 01.06 | 02.06 |
| [64] | **11.06** | Not Reported | Not Reported | Not Reported | **11.06** |
| [65] | **12.06** | 10.05 | Not Reported | **12.06** | 23.06 |
| [66] | **21.06** | 18.02 | **21.06** | 07.07 | 28.07 |
| [67] | **27.06** | Not Reported | Not Reported | Not Reported | **27.06** |
| [68] | **01.07** | Not Reported | Not Reported | **01.07** | 03.07 |
| [69] | **04.07** | 04.07 | **04.07** | 04.07 | 04.07 |
| [70] | **15.07** | Not Reported | Not Reported | Not Reported | **15.07** |
| [71] | **26.07** | 26.07 | **26.07** | 26.07 | 26.07 |
| [72] | **26.07** | Not Reported | Not Reported | Not Reported | **26.07** |
| [73] | **27.07** | 27.07 | **27.07** | 27.07 | 27.07 |
| [74] | **30.07** | 30.07 | **30.07** | 30.07 | 30.07 |
| [75] | **03.08** | 03.08 | **03.08** | 03.08 | 03.08 |
| [76] | **09.08** | 09.08 | **09.08** | 09.08 | 09.08 |
| [77] | **14.08** | 14.08 | **14.08** | 14.08 | 14.08 |
| [78] | **22.09** | 22.09 | Not Reported | **22.09** | 12.10 |

**Table S2.** Exluded studies with reasons.

| ID | Reason |
|---|---|
| [102] | Wrong Study Design |
| [103] | Wrong Patient Population |
| [104] | Wrong Target Condition |
| [105] | Wrong Outcomes |
| [106] | Wrong Outcomes |
| [107] | Wrong Outcomes |
| [108] | Wrong Outcomes |
| [109] | Wrong Outcomes |
| [110] | Wrong Outcomes |
| [111] | Wrong Outcomes |
| [112] | Wrong Outcomes |

**Table S3.** Full characteristics of included reviews.

| ID | No of interesting studies | No of models | Participants | | | | Control | | Index | | | | Outcomes (percentages) | Funding | Conflict of Interest |
|---|---|---|---|---|---|---|---|---|---|---|---|---|---|---|---|
| | | | No | COVID+ | Age | Sex | No | Type | Model | Data type | Data size | Explainablity | | | |
| | | | NR = Not Reported | | | | NR = Not Reported | 1 = Viral<br>2 = Bacterial<br>3 = Unknown<br>4 = Unclear<br>5 = Asymptomatic | NR = Not Reported<br>M = Multiple | 1 = X-ray<br>2 = CT (Computed Tomography)<br>3 = US (Ultrasound) | NR = Not Reported<br>R = Reported<br>CT = Computed Tomography | | Acc = Accuracy<br>AUC = Area Under Curve<br>Cindex = Concordance Index<br>F1 = F-score<br>NPV = Negative Predictive Value<br>Prec = Precision<br>Sens = Sensitivity<br>Spec = Specificity | NR = Not Reported<br>R = Reported<br>N = No Conflict of Interest | |
| [57] | 13 | 18 | 14,822 | 4,295 | NR | NR | 10,597 | 1, 2, 3, 4, 5 | M | 1, 2 | X-Rays = 5,941<br>CT = 4,356 | NR | Acc = (73.1; 98)<br>AUC = (95.2; 97.9)<br>Sens = (74; 100)<br>Spec = (67; 96) | R | NR |
| [58] | 5 | 26 | 1,028 | 394 | NR | NR | 634 | 5 | M | 1 | X-Rays = 1,028 | NR | Acc = (50; 98) | NR | NR |
| [59] | 12 | 13 | 14,992 | 4,939 | NR | NR | 9,896 | 1, 2, 3, 4, 5 | M | 1, 2, 3 | CT = 509 | NR | Acc = (86.7; 95)<br>AUC = (78; 99.6)<br>Sens = (90.7; 97.4)<br>Spec = (83.3; 92.2) | R | NR |
| [60] | 15 | 16 | 13,427 | 4,017 | NR | NR | 9,480 | 1, 2, 3, 4, 5 | M | 1, 2 | X-Rays = 7,499<br>CT = 10,022 | NR | Acc = 89.3<br>AUC = (94; 99)<br>Sens = (67; 100)<br>Spec = (76; 100) | NR | N |

| ID | No of interesting studies | No of models | Participants | | | | Control | | Index | | | | Outcomes (percentages) | Funding | Conflict of Interest |
|---|---|---|---|---|---|---|---|---|---|---|---|---|---|---|---|
| | | | No | COVID+ | Age | Sex | No | Type | Model | Data type | Data size | Explainablity | | | |
| | | | NR = Not Reported | | | | NR = Not Reported | 1 = Viral<br>2 = Bacterial<br>3 = Unknown<br>4 = Unclear<br>5 = Asymptomatic | NR = Not Reported<br>M = Multiple | 1 = X-ray<br>2 = CT (Computed Tomography)<br>3 = US (Ultrasound) | NR = Not Reported<br>R = Reported<br>CT = Computed Tomography | | Acc = Accuracy<br>AUC = Area Under Curve<br>Cindex = Concordance Index<br>F1 = F-score<br>NPV = Negative Predictive Value<br>Prec = Precision<br>Sens = Sensitivity<br>Spec = Specificity | NR = Not Reported<br>R = Reported<br>N = No Conflict of Interest | |
| [61] | 6 | 9 | 19,058 | 546 | NR | NR | 1,435 | 1, 4, 5 | M | 1, 2 | X-Rays= 16,756<br>CT = 6,039 | NR | Acc = (79.3; 98)<br>AUC = 97.91<br>Sens = (67; 94.06)<br>Spec = (83; 95.47) | NR | N |
| [62] | 1 | 1 | NR | NR | NR | NR | NR | 4 | NR | 1 | NR | NR | Sens = 85 | R | N |
| [63] | 1 | 1 | NR | NR | NR | NR | NR | 4 | NR | 1 | NR | NR | AUC = 96<br>Sens = 90<br>Spec = 96 | R | N |
| [64] | 1 | 1 | NR | NR | NR | NR | NR | 3 | NR | 2 | CT = 4,356 | NR | Sens = 90<br>Spec = 96 | R | N |
| [65] | 7 | 9 | NR | 160 | NR | NR | NR | 1, 2, 3, 4, 5 | M | 1, 2 | X-Rays = 150<br>CT = 768 | NR | Acc = (86.7; 99.68)<br>F1 = (91; 95.52) | NR | N |
| [66] | 10 | 15 | NR | NR | NR | NR | NR | 3, 4, 5 | M | 1, 2, 3 | NR | NR | Acc = (76; 100)<br>AUC = (81.9; 96)<br>Sens = 96.8 | R | N |
| [67] | 6 | 11 | 3,680 | 90 | NR | NR | 84 | 1, 2, 3, 4, 5 | M | 1, 2 | X-Rays = 1,427<br>CT = 4,536 | NR | Acc = (73.1; 98.3)<br>AUC = (78; 97)<br>NPV = (81; 88)<br>Sens = (67.1; 98.66)<br>Spec = (76.4; 96.46) | NR | NR |

| ID | No of interesting studies | No of models | Participants | | | | Control | | Index | | | | Outcomes (percentages) | Funding | Conflict of Interest |
|---|---|---|---|---|---|---|---|---|---|---|---|---|---|---|---|
| | | | No | COVID+ | Age | Sex | No | Type | Model | Data type | Data size | Explainablity | | | |
| | | | NR = Not Reported<br>Age = Range of mean ages<br>Sex = Range of percentage of men | | | | NR = Not Reported | 1 = Viral<br>2 = Bacterial<br>3 = Unknown<br>4 = Unclear<br>5 = Asymptomatic | NR = Not Reported<br>M = Multiple | 1 = X-ray<br>2 = CT (Computed Tomography)<br>3 = US (Ultrasound) | NR = Not Reported<br>R = Reported<br>CT = Computed Tomography | | Acc = Accuracy<br>AUC = Area Under Curve<br>Cindex = Concordance Index<br>F1 = F-score<br>NPV = Negative Predictive Value<br>Prec = Precision<br>Sens = Sensitivity<br>Spec = Specificity | NR = Not Reported<br>R = Reported<br>N = No Conflict of Interest | |
| [68] | 42 | 48 | 52,540 | 8,050 | (31, 71) | (35, 67) | 38,780 | 1, 2, 4, 5 | M | 1, 2, 3 | NR | NR | Prec = (35; 99)<br>NPV = (66; 97)<br>Sens = (79; 100)<br>Spec = (67; 100)<br>Cindex = (81-99.8) | R | N |
| [69] | 21 | 21 | 14,255 | 371 | NR | NR | 13,884 | 1, 2, 4, 5 | M | 1, 2 | X-Rays = 18,184<br>CT = 48,809 | NR | Acc = (73.1; 99.6)<br>Sens = (74; 100)<br>Spec = (67; 96) | R | NR |
| [70] | 8 | 8 | 13,870 | NR | NR | NR | NR | 4, 5 | NR | 1, 2 | X-Rays = 13,975<br>CT = 630 | NR | Acc = (82.9; 99.68)<br>AUC = (99.4; 99.6)<br>Prec = 84<br>NPV = 98.2<br>Sens = (84; 98.93)<br>Spec = (80.5; 97.6) | R | NR |
| [71] | 67 | 68 | NR | NR | NR | NR | NR | 1, 2, 3, 4, 5 | M | 1, 2 | X-Rays = 299,267<br>CT = 84,317 | R | Acc = (73.1; 99.8)<br>AUC = (31; 99.8)<br>F1 = 89 | R | NR |
| [72] | 12 | 18 | 19,607 | NR | NR | NR | NR | 4 | M | 1, 2 | X-Rays = 25,391<br>CT = 51 | NR | Acc = (87; 99.68)<br>F1=97.8<br>Prec = (96.4; 99.7)<br>Sens = (87; 98.66)<br>Spec = 87.9 | NR | N |

| ID | No of interesting studies | No of models | Participants | | | | Control | | Index | | | | Outcomes (percentages) | Funding | Conflict of Interest |
|---|---|---|---|---|---|---|---|---|---|---|---|---|---|---|---|
| | | | No | COVID+ | Age | Sex | No | Type | Model | Data type | Data size | Explainablity | | | |
| | | | NR = Not Reported | | | | NR = Not Reported | 1 = Viral<br>2 = Bacterial<br>3 = Unknown<br>4 = Unclear<br>5 = Asymptomatic | NR = Not Reported<br>M = Multiple | 1 = X-ray<br>2 = CT (Computed Tomography)<br>3 = US (Ultrasound) | NR = Not Reported<br>R = Reported<br>CT = Computed Tomography | | Acc = Accuracy<br>AUC = Area Under Curve<br>Cindex = Concordance Index<br>F1 = F-score<br>NPV = Negative Predictive Value<br>Prec = Precision<br>Sens = Sensitivity<br>Spec = Specificity | NR = Not Reported<br>R = Reported<br>N = No Conflict of Interest | |
| [73] | 106 | 116 | 187,976 | 18,882 | NR | NR | 122,248 | 1, 2, 3, 4, 5 | M | 1, 2 | X-Rays = 352,377<br>CT = 179,951 | R | Acc = (71.92; 100)<br>AUC = (70; 99.84)<br>F1 = (64; 100)<br>Prec = (69.89; 100)<br>Sens = (68.91; 100)<br>Spec = (61.5; 100) | NR | NR |
| [74] | 12 | 13 | 19,572 | 789 | NR | NR | 2,072 | 1, 2, 3, 4, 5 | M | 1, 2 | X-Rays = 25,743<br>CT = 8,755 | NR | Acc = (79.3; 98.3 )<br>AUC = (95.4; 99.6)<br>Sens = (67; 97.91)<br>Spec = (83; 92.2) | NR | NR |
| [75] | 10 | 12 | 256 | NR | NR | NR | NR | 3, 4, 5 | M | 1, 2, 3 | CT = 603 | NR | Acc = (73.1; 100)<br>AUC = (96; 99.6)<br>F1 = (89; 100)<br>Prec = 97.63<br>Sens = (74; 100)<br>Spec = (67; 100) | NR | NR |
| [76] | 45 | 45 | NR | NR | NR | NR | NR | 1, 2, 3, 4, 5 | M | 1, 2 | X-Rays = 463,019<br>CT = 150,989 | NR | Acc = (76; 100)<br>AUC = (81; 100)<br>F1 = (63; 100)<br>Prec = (35.27; 100)<br>NPV = (90; 100)<br>Sens = (80; 100)<br>Spec = (61.5; 100) | NR | NR |

| ID | No of interesting studies | No of models | Participants | | | | Control | | Index | | | | Outcomes (percentages) | Funding | Conflict of Interest |
|---|---|---|---|---|---|---|---|---|---|---|---|---|---|---|---|
| | | | No | COVID+ | Age | Sex | No | Type | Model | Data type | Data size | Explainablity | | | |
| | | | NR = Not Reported | | | | NR = Not Reported | 1 = Viral<br>2 = Bacterial<br>3 = Unknown<br>4 = Unclear<br>5 = Asymptomatic | NR = Not Reported<br>M = Multiple | 1 = X-ray<br>2 = CT (Computed Tomography)<br>3 = US (Ultrasound) | NR = Not Reported<br>R = Reported<br>CT = Computed Tomography | | Acc = Accuracy<br>AUC = Area Under Curve<br>Cindex = Concordance Index<br>F1 = F-score<br>NPV = Negative Predictive Value<br>Prec = Precision<br>Sens = Sensitivity<br>Spec = Specificity | NR = Not Reported<br>R = Reported<br>N = No Conflict of Interest | |
| [77] | 24 | 29 | 3,238 | 1,650 | NR | NR | 1,578 | 1, 2, 3, 4, 5 | M | 1, 2 | X-Rays = 32,840<br>CT = 149,078 | NR | Acc = (64.4; 99.87)<br>AUC = (67; 100)<br>F1 = (70; 99.8)<br>Prec = (89.8; 100)<br>NPV=100<br>Sens = (61; 100)<br>Spec = (28; 100) | R | N |
| [78] | 27 | 31 | 21,053 | 2,570 | NR | NR | 1,678 | 1, 2, 3, 4, 5 | M | 1, 2 | X-Rays = 194,470<br>CT = 55,245 | NR | Acc = (73.1; 99.68)<br>AUC (92; 99.6)<br>F1 = (87; 97.5)<br>NPV = (84; 93.36)<br>Sens = (8; 100)<br>Spec = (67; 99.99) | R | NR |

**Table S4.** In-depth characteristics of primary studies.

| | 73 primary studies (included in one review only) | | | 138 primary studies (included in at least one review) | | | | | |
|---|---|---|---|---|---|---|---|---|---|
| | | | | Analysis A | | | Analysis B | | |
| Variable | N (%) | Mean (range) | NOS | N (%) | Mean (range) | NOS | N (%) | Mean (range) | NOS |
| Number of participants | | | | | | | | | |
| Total | 62,710 | 2,613 (60-16,340) | 24 | 63,535 | 2,444 (60-16,340) | 26 | 174,277 | 2,954 (45-32,717) | 59 |
| COVID-19 | 10,720 (17%) | 466 (33-3,389) | 23 | 10,766 (17%) | 449 (33-3,389) | 24 | 19,170 (11%) | 369 (33-3,389) | 52 |
| Control | 51,523 (83%) | 3,037 (25-16,232) | 17 | 52,002 (83%) | 2,737 (25-16,232) | 19 | 108,268 (62%) | 2,578 (25-23,664) | 42 |
| CT scans | | | | | | | | | |
| Total | 45,942 | 3,282 (10-21,658) | 47 | 45,942 | 3,282 (10-21,658) | 70 | 282,029 | 9,401 (10-132,583) | 96 |
| COVID-19 | 29,261 (64%) | 2.251 (10-21,658) | 46 | 29,411 (64%) | 2,101 (10-21,658) | 71 | 134,312 (48%) | 4,631 (10-65,806) | 95 |
| X-Ray images | | | | | | | | | |
| Total | 586,489 | 22,557 (40-339,271) | 53 | 613,747 | 21,164 (40-339,271) | 77 | 813,633 | 18,922 (40-339,271) | 102 |
| COVID-19 | 7,172 (1%) | 276 (25-4,273) | 53 | 8858 (1%) | 286 (25-1,565) | 77 | 15,759 (2%) | 343 (25-4,273) | 106 |
| Other images | | | | | | | | | |
| Total | 6,087 | 6,087 (6,087-6,087) | 64 | 6,087 | 6,087 (6,087-6,087) | 106 | 6,287 | 3,144 (200-6,087) | 129 |
| COVID-19 | 231 (4%) | 231 (231-231) | 64 | 231 (4%) | 231 (231-231) | 107 | 231 (4%) | 231 (231-231) | 128 |
| Comparison | | | | | | | | | |
| Bacterial pneumonia | 8 (11%) | NA | 73 | 10 (9%) | NA | 131 | 10 (9%) | NA | 131 |
| Viral pneumonia | 9 (12%) | NA | 73 | 11 (10%) | NA | 129 | 11 (10%) | NA | 129 |
| Unknown pneumonia | 18 (25%) | NA | 73 | 21 (19%) | NA | 107 | 21 (19%) | NA | 108 |
| Unclear | 21 (29%) | NA | 73 | 32 (23%) | NA | 93 | 32 (23%) | NA | 93 |
| Asymptomatic controls | | | 73 | | | 92 | | | 92 |
| Healthy (normal) | 23 (32%) | NA | | 27 (20%) | NA | | 27 (20%) | NA | |
| Non-COVID-19 (negative cases) | 18 (25%) | NA | | 21 (15%) | NA | | 21 (15%) | NA | |
| Other | 2 (3%) | NA | | 2 (1%) | NA | | 2 (1%) | NA | |

NA = Not Applicable, NOS = Number of Studies, CT = Computed Tomography, CAM = Class Activation Mapping, Grad-CAM = Gradient-weighted Class Activation Mapping, LIME = Local Interpretable Model-agnostic Explanations, T-SNE = t-distributed stochastic neighbour embedding

| | 73 primary studies (included in one review only) | | | 138 primary studies (included in at least one review) | | | | | |
|---|---|---|---|---|---|---|---|---|---|
| | | | | Analysis A | | | Analysis B | | |
| Variable | N (%) | Mean (range) | NOS | N (%) | Mean (range) | NOS | N (%) | Mean (range) | NOS |
| Datasets used | | | 19 | | | 21 | | | 21 |
| Kaggle's Chest X-Ray Image (Pneumonia) (also dataset, repository) | 6 (7%) | NA | | 6 (4%) | NA | | 6 (4%) | NA | |
| COVID-19 X-ray image database | 4 (5%) | NA | | 4 (3%) | NA | | 4 (3%) | NA | |
| COVIDx (also X-ray 5k) | 2 (2%) | NA | | 3 (2%) | NA | | 3 (2%) | NA | |
| Chest X-Ray Images (Pneumonia) | 1 (1%) | NA | | 1 (1%) | NA | | 1 (1%) | NA | |
| Cohen (also JP, Joseph Paul) | 1 (1%) | NA | | 1 (1%) | NA | | 1 (1%) | NA | |
| Other | 14 (17%) | NA | | 20 (13%) | NA | | 20 (13%) | NA | |
| Architecture | | | 52 | | | 66 | | | 66 |
| ResNet (also Res2Net, 101, 152, 18, 23, 50, 50V2) | 13 (14%) | NA | | 13 (8%) | NA | | 13 (8%) | NA | |
| DenseNet (also 121, 161, 169, 201, 264) | 12 (13%) | NA | | 14 (9%) | NA | | 14 (9%) | NA | |
| VGG (also 16, 19) | 8 (9%) | NA | | 18 (11%) | NA | | 18 (11%) | NA | |
| Inception (also V1, V2, V3, V4) | 6 (6%) | NA | | 6 (4%) | NA | | 6 (4%) | NA | |
| GoogleNet | 3 (3%) | NA | | 3 (2%) | NA | | 3 (2%) | NA | |
| AlexNet | 2 (2%) | NA | | 2 (1%) | NA | | 2 (1%) | NA | |
| Xception | 2 (2%) | NA | | 2 (1%) | NA | | 2 (1%) | NA | |
| MobileNet (also V2) | 1 (1%) | NA | | 2 (1%) | NA | | 2 (1%) | NA | |
| SqueezeNet | 1 (1%) | NA | | 1 (1%) | NA | | 1 (1%) | NA | |
| UNet (also +, ++) | 1 (1%) | NA | | 2 (1%) | NA | | 2 (1%) | NA | |
| Other | 22 (24%) | NA | | 22 (14%) | NA | | 22 (14%) | NA | |

NA = Not Applicable, NOS = Number of Studies, CT = Computed Tomography, CAM = Class Activation Mapping, Grad-CAM = Gradient-weighted Class Activation Mapping, LIME = Local Interpretable Model-agnostic Explanations, T-SNE = t-distributed stochastic neighbour embedding

| | 73 primary studies (included in one review only) | | | 138 primary studies (included in at least one review) | | | | | |
|---|---|---|---|---|---|---|---|---|---|
| | | | | Analysis A | | | Analysis B | | |
| Variable | N (%) | Mean (range) | NOS | N (%) | Mean (range) | NOS | N (%) | Mean (range) | NOS |
| Metrics | | | | | | | | | |
| Accuracy | 51 (70%) | 94.02 (71.92-100) | 51 | 68 (49%) | 93.88 (71.92-100) | 68 | 93 (67%) | 93.81 (71.92-100) | 93 |
| Sensitivity | 44 (60%) | 90.93 (68.91-100) | 44 | 47 (34%) | 91.19 (68.91-100) | 47 | 75 (54%) | 92.32 (68.91-100) | 75 |
| Specificity | 33 (45%) | 92.82 (78.57-100) | 34 | 37 (27%) | 92.16 (78.57-100) | 38 | 49 (36%) | 90.65 (28-100) | 50 |
| F-score | 24 (33%) | 89.85 (63.13-100) | 24 | 27 (20%) | 89.87 (69.13-100) | 27 | 46 (33%) | 90.96 (69.13-100) | 46 |
| Precision | 22 (30%) | 93.92 (69.89-100) | 22 | 23 (17%) | 93.91 (69.89-100) | 23 | 49 (36%) | 92.89 (54-100) | 49 |
| Area Under Curve | 18 (25%) | 96.27 (90-99.99) | 18 | 26 (19%) | 94.58 (70-99.99) | 26 | 44 (32%) | 94.77 (67-99.99) | 44 |
| Negative Predictive Value | 1 (1%) | 94.50 (94.50-94.50) | 1 | 1 (1%) | 94.50 (94.50-94.50) | 1 | 7 (5%) | 93.17 (66-100) | 7 |
| Balanced Accuracy | 0 (0%) | NA | 0 | 0 (0%) | NA | 0 | 1 (1%) | 66 (66-66) | 1 |
| Other | 11 (15%) | NA | 11 | 12 (9%) | NA | 12 | 21 (15%) | NA | 21 |
| Post-processing | | | 21 | | | 36 | | | 39 |
| Grad-CAM (also ++) | 6 (25%) | NA | | 6 (17%) | NA | | 8 (20%) | NA | |
| CAM | 3 (13%) | NA | | 3 (9%) | NA | | 3 (8%) | NA | |
| CAM/Grad-CAM (unclear) | 2 (8%) | NA | | 14 (40%) | NA | | 15 (38%) | NA | |
| T-SNE | 2 (8%) | NA | | 2 (6%) | NA | | 2 (5%) | NA | |
| Saliency Maps | 1 (4%) | NA | | 1 (3%) | NA | | 1 (3%) | NA | |
| LIME | 1 (4%) | NA | | 1 (3%) | NA | | 1 (3%) | NA | |
| Other | 11 (46%) | NA | | 8 (23%) | NA | | 10 (25%) | NA | |

NA = Not Applicable, NOS = Number of Studies, CT = Computed Tomography, CAM = Class Activation Mapping, Grad-CAM = Gradient-weighted Class Activation Mapping, LIME = Local Interpretable Model-agnostic Explanations, T-SNE = t-distributed stochastic neighbour embedding

**Table S5.** Non-overlapping of in-depth characteristics variables.

| | 138 primary studies (included in at least one review) | |
|---|---|---|
| | Analysis A | Analysis B |
| Variable | N (%) | N (%) |
| Number of participants | | |
| Total | 45 (33%) | 12 (9%) |
| COVID-19 | 37 (27%) | 9 (7%) |
| Control | 33 (24%) | 10 (7%) |
| CT scans | | |
| Total | 38 (28%) | 12 (9%) |
| COVID-19 | 34 (25%) | 10 (7%) |
| X-Ray images | | |
| Total | 40 (29%) | 15 (11%) |
| COVID-19 | 39 (28%) | 10 (7%) |
| Other images | | |
| Total | 23 (17%) | 0 (0%) |
| COVID-19 | 21 (15%) | 0 (0%) |
| Comparison | | |
| Bacterial pneumonia | 7 (5%) | 7 (5%) |
| Viral pneumonia | 9 (7%) | 9 (7%) |
| Unknown pneumonia | 31 (22%) | 30 (22%) |
| Unclear | 45 (33%) | 45 (33%) |
| Asymptomatic controls | 44 (32%) | 44 (32%) |
| Datasets used | 54 (36%) | 54 (36%) |
| Architecture | 45 (33%) | 45 (33%) |
| Metrics | | |
| Accuracy | 39 (28%) | 14 (10%) |
| Sensitivity | 45 (33%) | 17 (12%) |
| Specificity | 28 (20%) | 16 (12%) |
| F-score | 24 (17%) | 5 (4%) |
| Precision | 31 (22%) | 5 (4%) |
| Area Under Curve | 26 (19%) | 8 (6%) |
| Negative Predictive Value | 6 (4%) | 0 (0%) |
| Balanced Accuracy | 1 (1%) | 0 (0%) |
| Other | 11 (8%) | 2 (1%) |
| Post processing | 9 (7%) | 3 (2%) |

CT = Computed Tomography

**Table S6.** Non-reporting of in-depth characteristics variables.

| | 73 primary studies (included in one review only) | 138 primary studies (included in at least one review) |
|---|---|---|
| Variable | N (%) | N (%) |
| Number of participants | | |
| Total | 49 (67%) | 67 (49%) |
| COVID-19 | 50 (68%) | 77 (56%) |
| Control | 56 (77%) | 86 (62%) |
| CT scans | | |
| Total | 26 (36%) | 30 (22%) |
| COVID-19 | 27 (37%) | 33 (24%) |
| X-Ray images | | |
| Total | 20 (27%) | 21 (15%) |
| COVID-19 | 20 (27%) | 22 (16%) |
| Other images | | |
| Total | 9 (12%) | 9 (7%) |
| COVID-19 | 9 (12%) | 10 (7%) |
| Datasets used | 54 (66%) | 63 (41%) |
| Architecture | 22 (24%) | 28 (18%) |
| Metrics | | |
| Accuracy | 22 (30%) | 31 (22%) |
| Sensitivity | 29 (40%) | 46 (33%) |
| Specificity | 39 (53%) | 72 (52%) |
| F-score | 49 (67%) | 87 (63%) |
| Precision | 51 (70%) | 84 (61%) |
| Area Under Curve | 55 (75%) | 86 (62%) |
| Negative Predictive Value | 72 (99%) | 131 (95%) |
| Balanced Accuracy | 73 (100%) | 137 (99%) |
| Other | 62 (85%) | 115 (83%) |
| Post processing | 52 (71%) | 95 (69%) |

CT = Computed Tomography

On next pages, COVID-19 diagnostic metrics are presented in Figures S1, S2, S3, S4, S5, and S6. Larger size of the bubble represents greater sample size of CT scans, X-Ray images and patients, accordingly. Darker colour represents more CT scans of, X-Ray images of and patients with COVID-19.

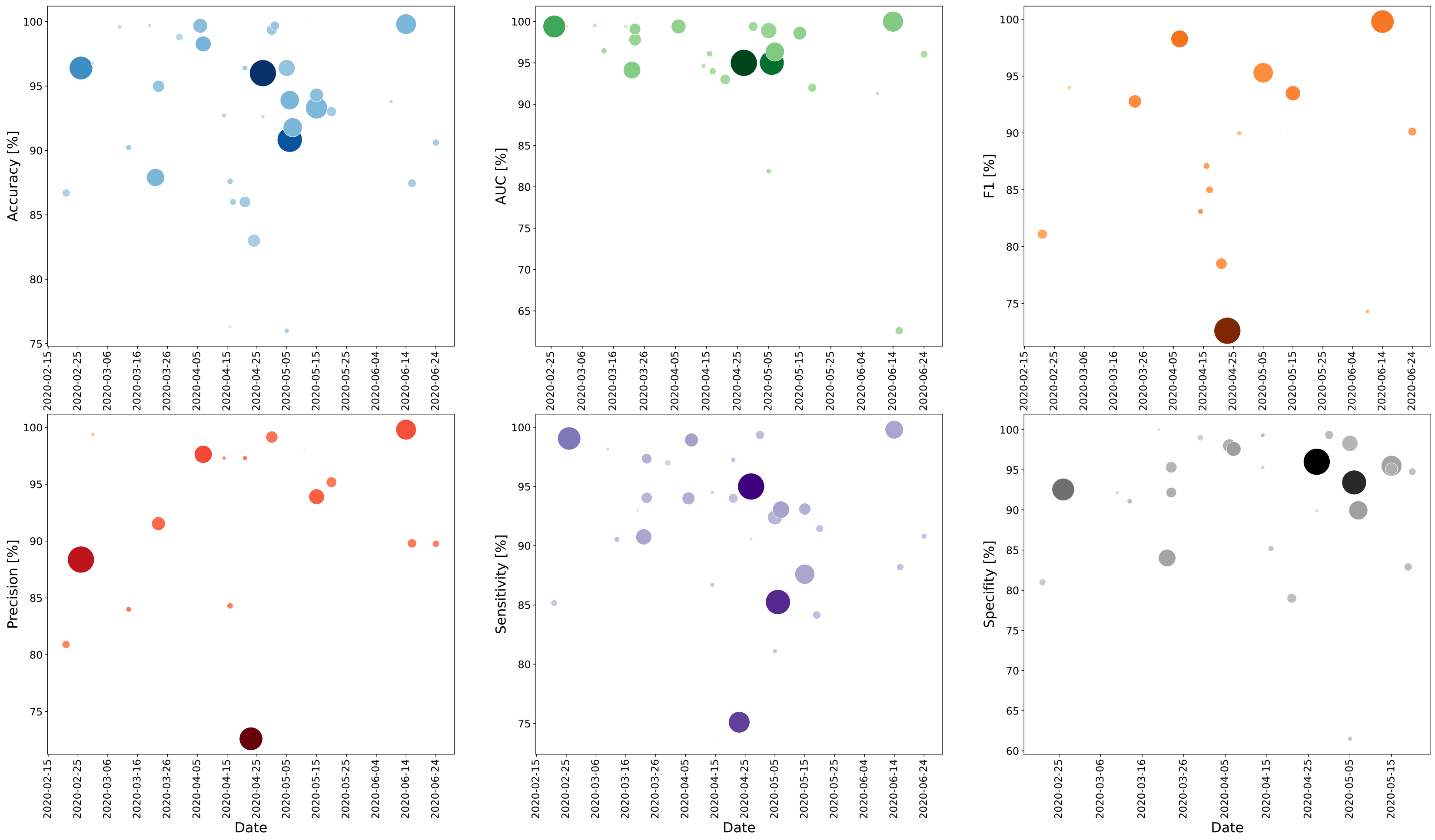


**Figure S1.** CT-based COVID-19 diagnosis, without imputation.

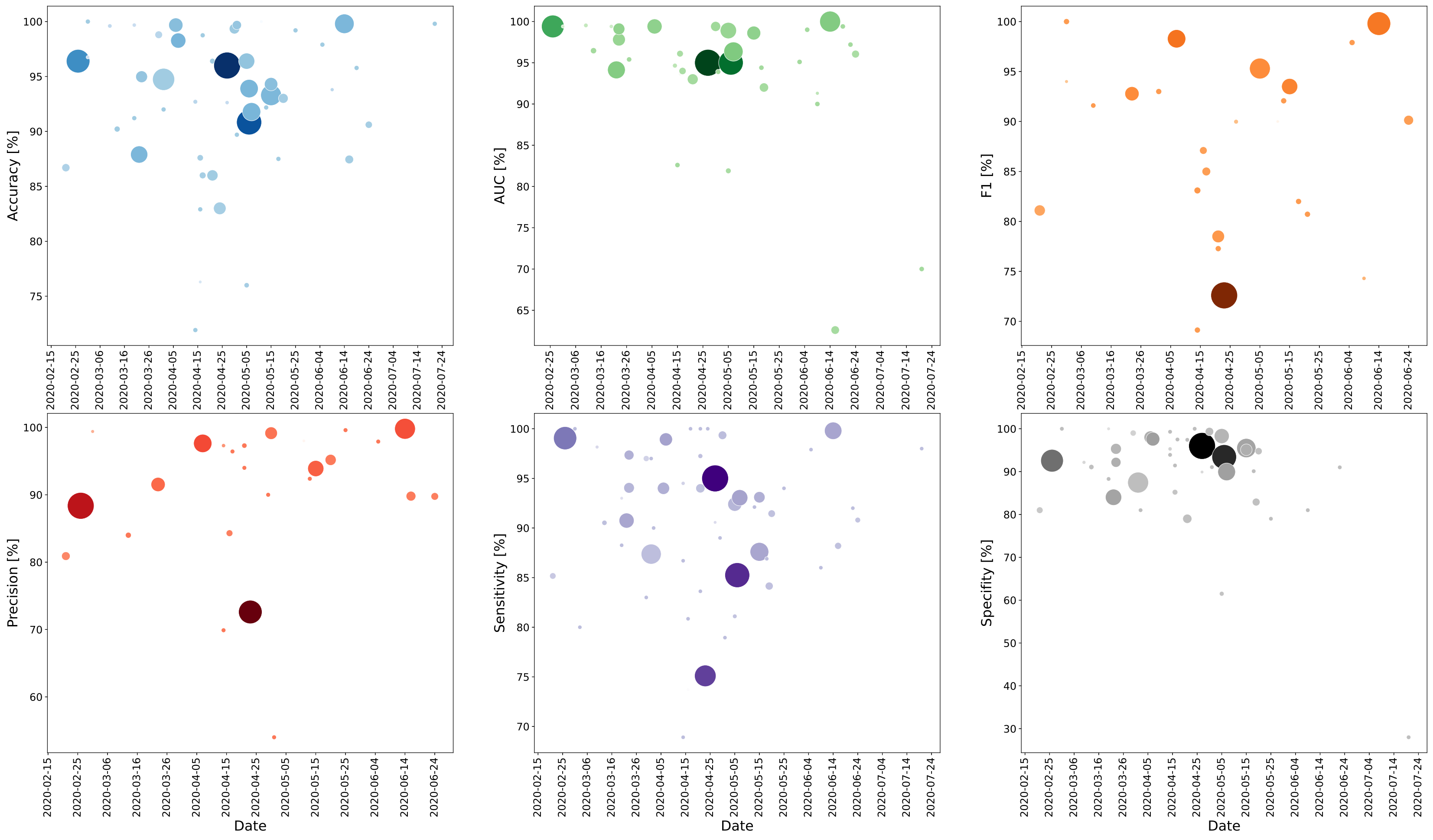


**Figure S2.** CT-based COVID-19 diagnosis, with modal imputation.

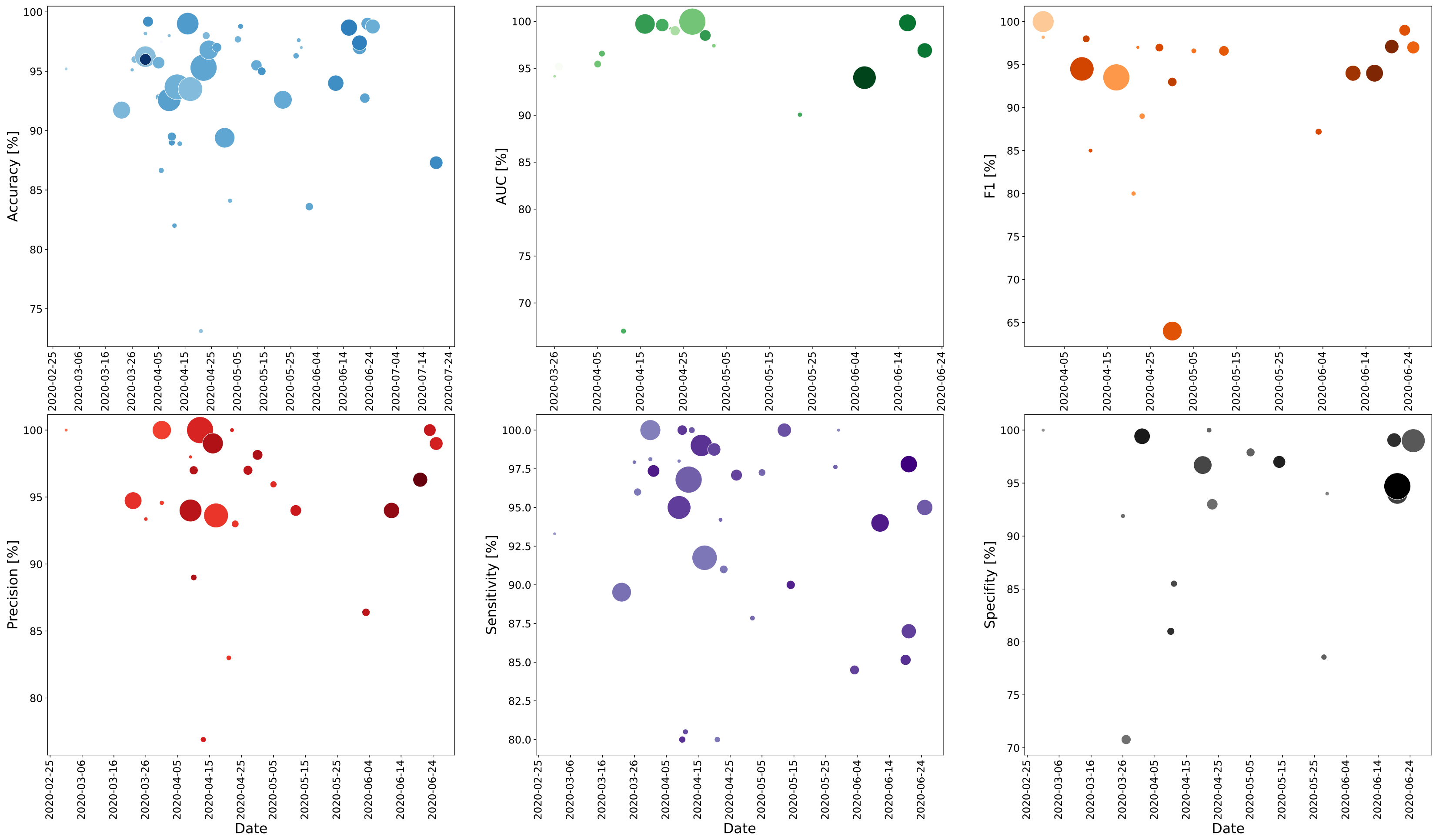


**Figure S3.** X-Ray-based COVID-19 diagnosis, without imputation.

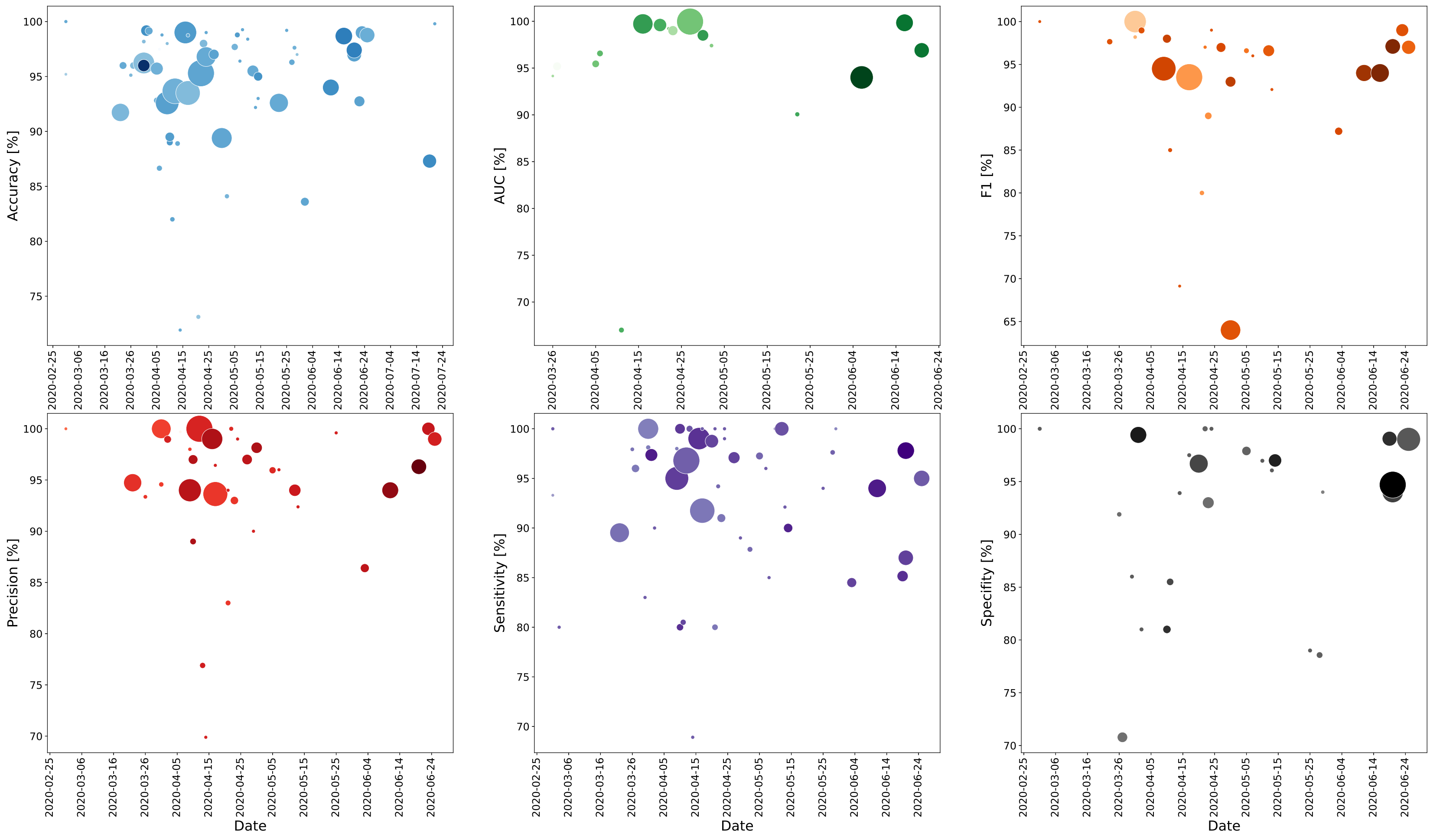


**Figure S4.** X-Ray-based COVID-19 diagnosis, with modal imputation.

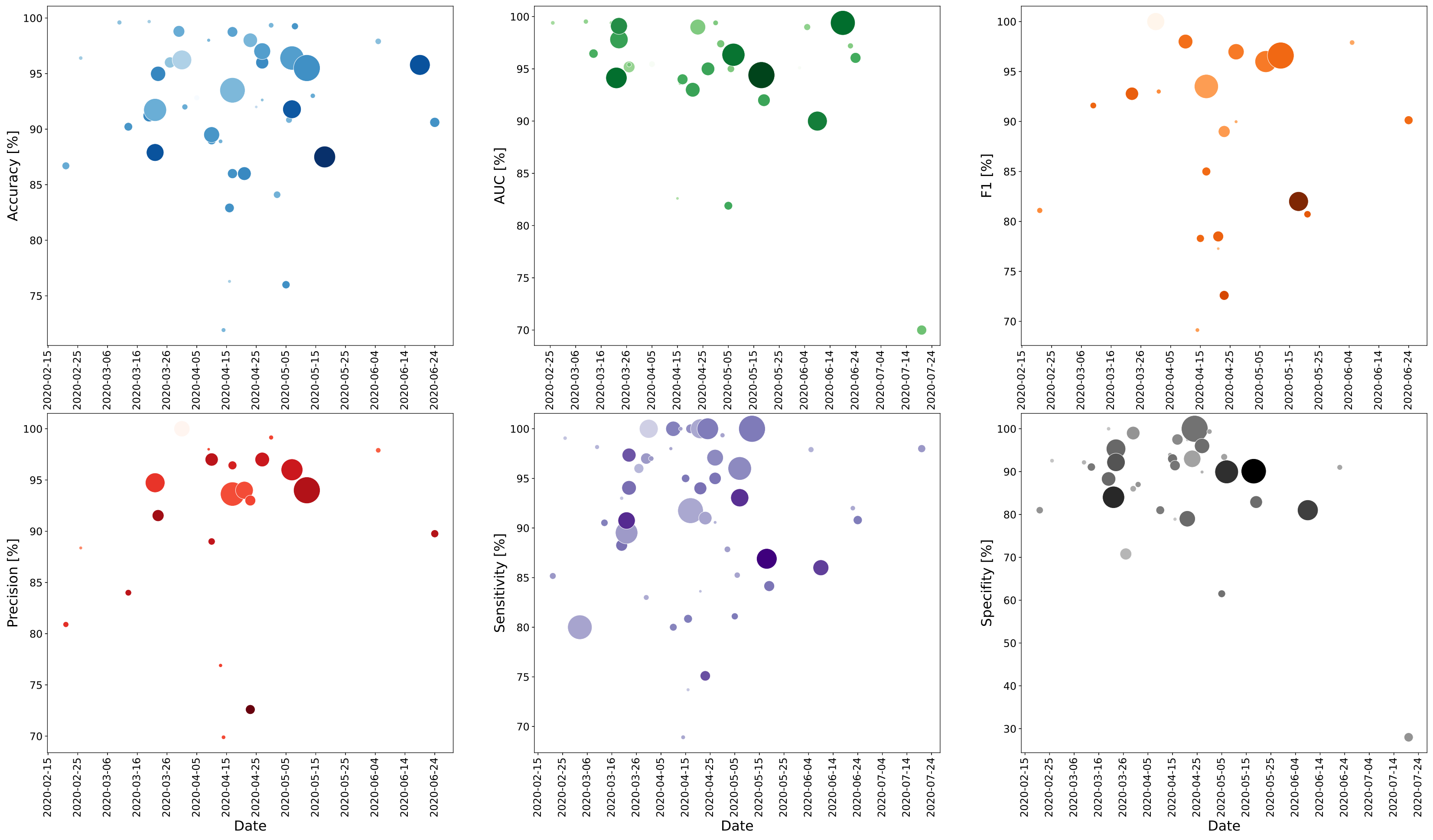


**Figure S5.** COVID-19 diagnosis with full patients number data provided, without imputation.

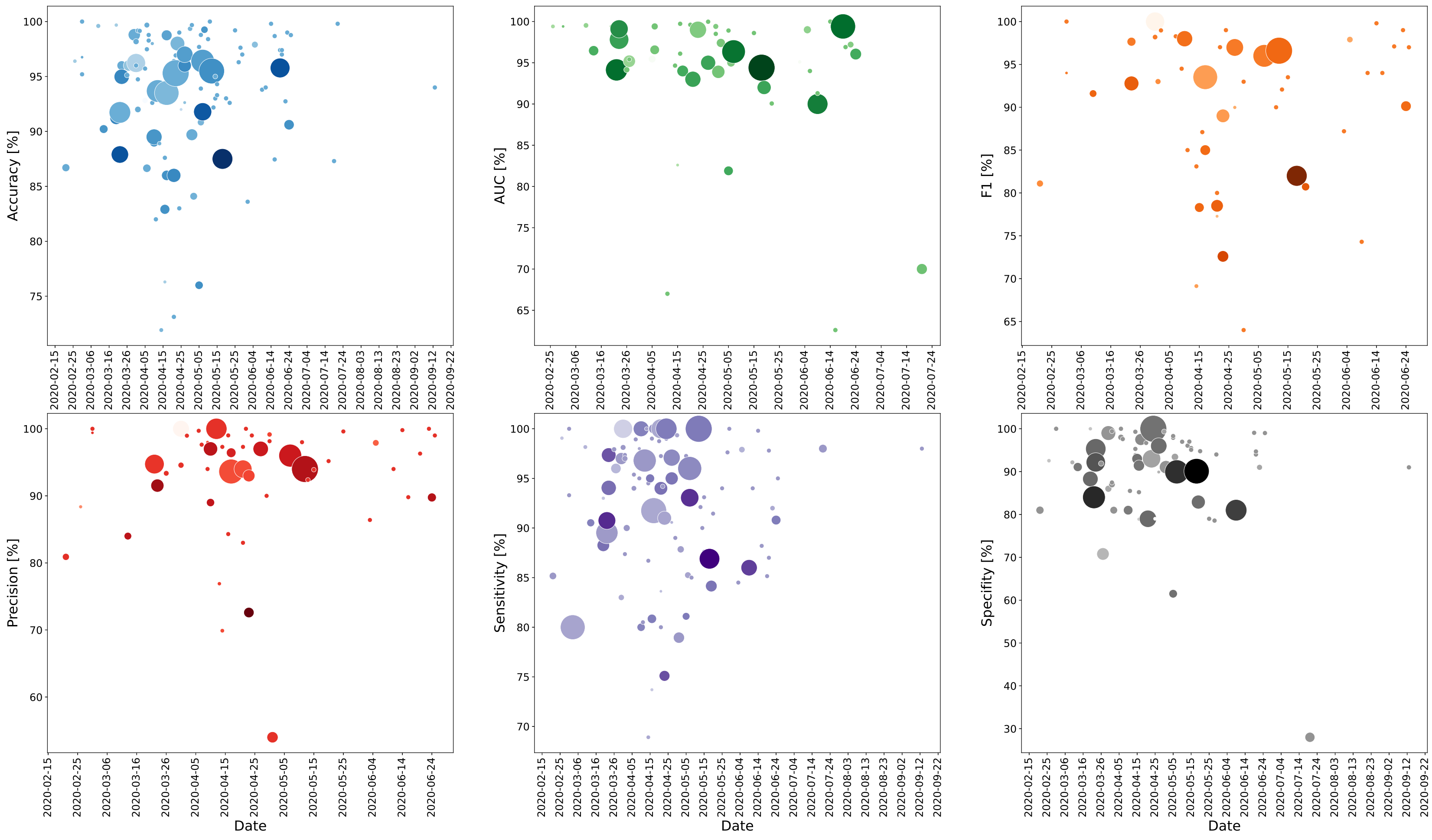


**Figure S6.** COVID-19 diagnosis with any patients number data provided, with modal imputation.

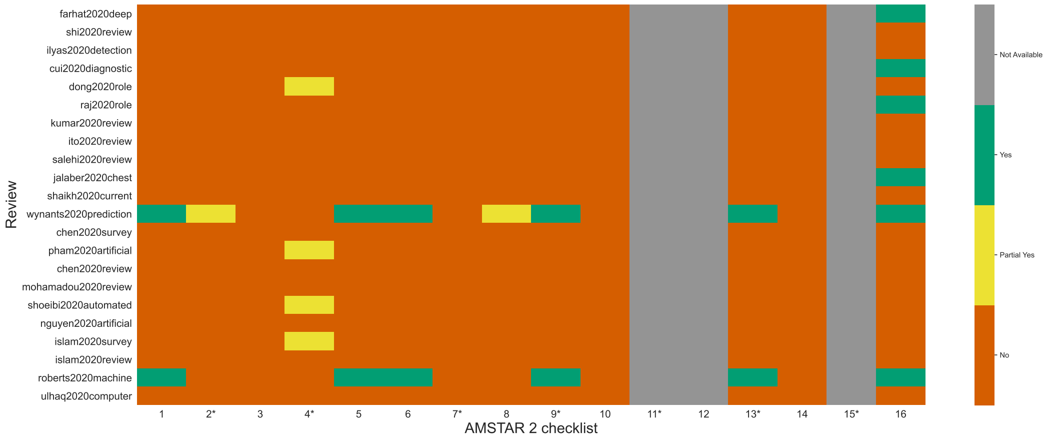


**Figure S7.** Review authors' judgements about each AMSTAR 2 item across all included studies; * denotes critical items.

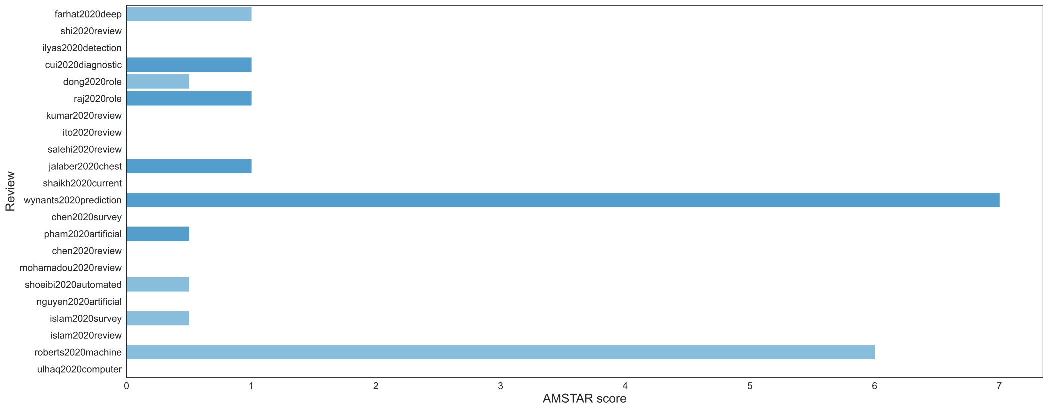


**Figure S8.** AMSTAR 2 score in each included review.

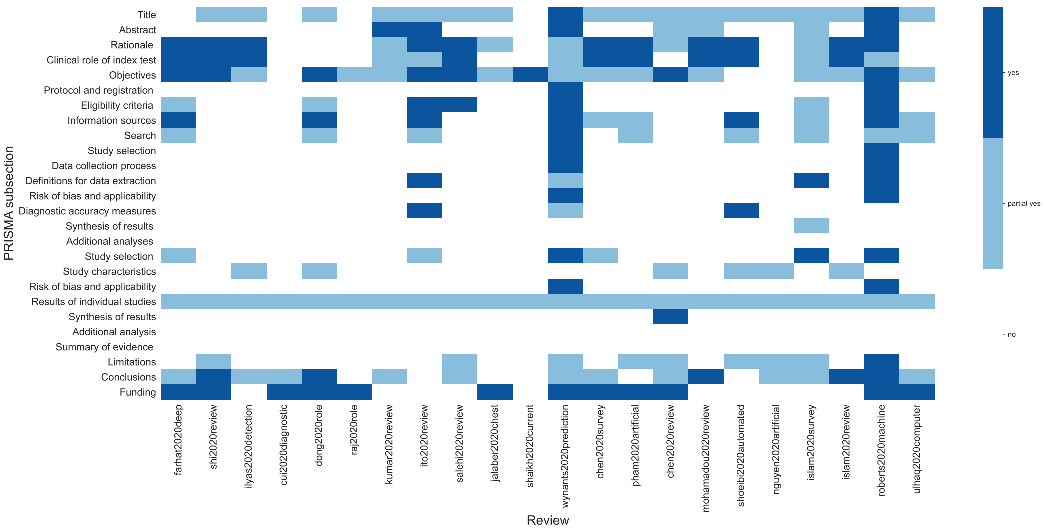


**Figure S9.** Review authors' judgements about each PRISMA-DTA item across all included studies.

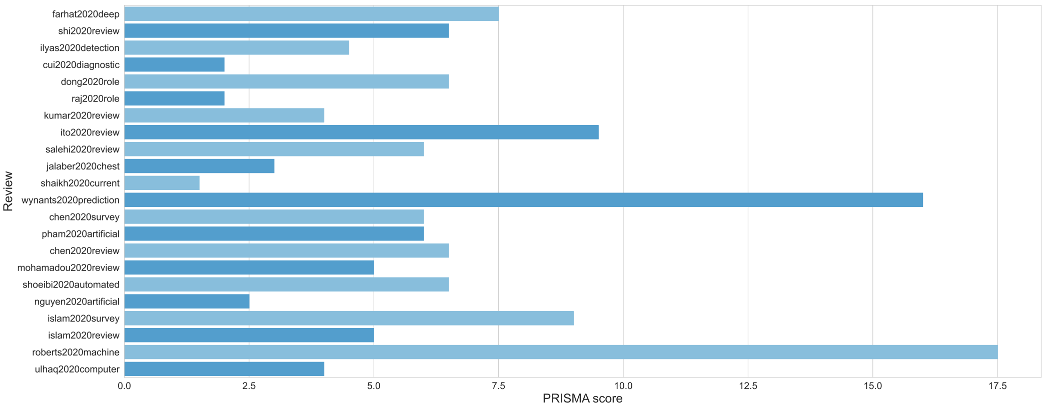


**Figure S10.** PRISMA-DTA score in each included review.

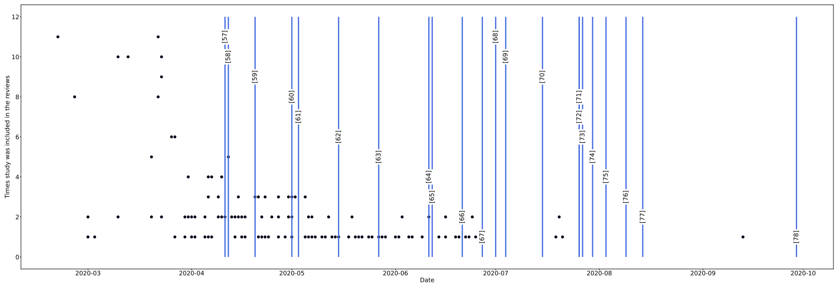


**Figure S11.** Appearance of included reviews (vertical lines, reference date, see Table S1 and interesting primary studies (dots).

**Table S7.** Time and resource wasting statistics.

| | A | B=A/X | C=A/Y | D | E | E/165 | F=D/E | G=D/X | H=D/Y | I=D/A | J | K |
|---|---|---|---|---|---|---|---|---|---|---|---|---|
| [57] | 13 | 13/26=50 | 13/52=25 | 13 | 13 | 7.88 | 13/13=100 | 13/26=50 | 13/52=25 | 100 | NA | NA |
| [58] | 5 | 5/26=19.23 | 5/53=9.43 | 3 | 16 | 9.79 | 3/16=18.75 | 3/26=11.54 | 3/53=5.67 | 3/5=60 | 0 | 0 |
| [59] | 12 | 12/59=20.34 | 12/72=16.67 | 3 | 19 | 11.51 | 3/19=15.79 | 3/59=5.08 | 3/72=4.17 | 3/12=25 | 0 | 0 |
| [60] | 15 | 15/24=62.50 | 15/106=14.15 | 1 | 20 | 12.12 | 1/20=5 | 1/24=4.17 | 1/106=0.94 | 1/15=6.67 | 0 | 0 |
| [61] | 6 | 6/21=28.57 | 6/107=5.61 | 0 | 20 | 12.12 | 0/20=0 | 0/21=0 | 0/106=0 | 0/6=0 | 0 | 1 |
| [62] | 1 | 1/116=0.86 | 1/127=0.79 | 1 | 21 | 12.73 | 1/21=4.76 | 1/116=0.86 | 1/127=0.79 | 1/1=100 | 0 | 0 |
| [63] | 1 | 1/10=10 | 1/136=0.73 | 0 | 21 | 12.73 | 0/21=0 | 0/10=0 | 0/136=0 | 0/1=0 | 0 | 0 |
| [64] | 1 | 1/10=10 | 1/147=0.68 | 0 | 21 | 12.73 | 0/21=0 | 0/10=0 | 0/147=0 | 0/1=0 | 0 | 0 |
| [65] | 7 | 7/32=21.88 | 7/147=4.76 | 1 | 22 | 13.33 | 1/22=4.54 | 1/32=3.13 | 1/147=0.68 | 1/7=14.29 | 0 | 0 |
| [66] | 10 | 10/113=8.85 | 10/155=6.45 | 9 | 31 | 18.79 | 9/31=29.03 | 9/113=7.96 | 9/155=5.81 | 9/10=90 | 3 | 4 |
| [67] | 6 | 6/59=10.17 | 6/160=3.75 | 1 | 32 | 19.39 | 1/32=3.13 | 1/59=1.69 | 1/160=0.63 | 1/6=16.67 | 1 | 1 |
| [68] | 42 | 42/107=39.25 | 42/161=26.09 | 27 | 59 | 35.76 | 27/59=45.76 | 27/107=25.23 | 27/160=16.88 | 27/42=64.29 | 0 | 0 |
| [69] | 21 | 21/139=15.11 | 21/161=13.04 | 2 | 61 | 36.97 | 2/61=3.28 | 2/139=1.43 | 2/161=1.24 | 2/21=9.52 | 0 | 0 |
| [70] | 8 | 8/47=17.02 | 8/161=4.97 | 1 | 62 | 37.58 | 1/62=1.61 | 1/47=2.13 | 1/161=0.62 | 1/8=12.5 | 0 | 0 |
| [71] | 67 | 67/164=40.85 | 67/164=40.85 | 37 | 99 | 60 | 37/99=37.37 | 37/164=22.56 | 37/164=22.56 | 37/67=55.22 | 4 | 7 |
| [72] | 12 | 12/67=17.91 | 12/164=7.32 | 2 | 101 | 61.21 | 2/101=1.98 | 2/67=2.99 | 2/164=1.22 | 2/12=16.67 | 2 | 2 |
| [73] | 106 | 106/163=65.03 | 106/164=64.63 | 45 | 146 | 88.48 | 45/146=30.82 | 45/163=27.61 | 45/164=27.44 | 45/106=42.45 | 0 | 0 |
| [74] | 12 | 12/139=8.63 | 12/164=7.32 | 1 | 147 | 89.09 | 1/147=0.68 | 1/139=0.72 | 1/164=0.61 | 1/12=8.33 | 1 | 1 |
| [75] | 10 | 10/71=14.08 | 10/164=6.10 | 1 | 148 | 89.70 | 1/148=0.68 | 1/71=1.41 | 1/164=0.61 | 1/10=10 | 1 | 1 |
| [76] | 45 | 45/125=36.00 | 45/164=27.44 | 9 | 157 | 95.15 | 9/157=5.73 | 9/125=7.2 | 9/164=5.49 | 9/45=20 | 4 | 5 |
| [77] | 24 | 24/163=14.72 | 24/164=14.63 | 7 | 164 | 99.39 | 7/164=4.27 | 7/163=4.29 | 7/164=4.27 | 7/24=29.17 | 1 | 1 |
| [78] | 27 | 27/165=16.36 | 27/165=16.36 | 1 | 165 | 100 | 1/165=0.61 | 1/165=0.61 | 1/165=0.61 | 1/27=3.70 | 0 | 0 |

**A** – number of primary studies included in the review (see Figure 4; **D** – number of included primary papers that were introduced (see Figure 3); **E** – cumulative sum of included primary papers that were introduced; **J** – cited reviews that were published; **K** – cited reviews both published and available as preprints; **X** – all primary papers available to the date of publishing last included study; **Y** – all primary papers available to the reference date (see Table S1, see Figure 4)).

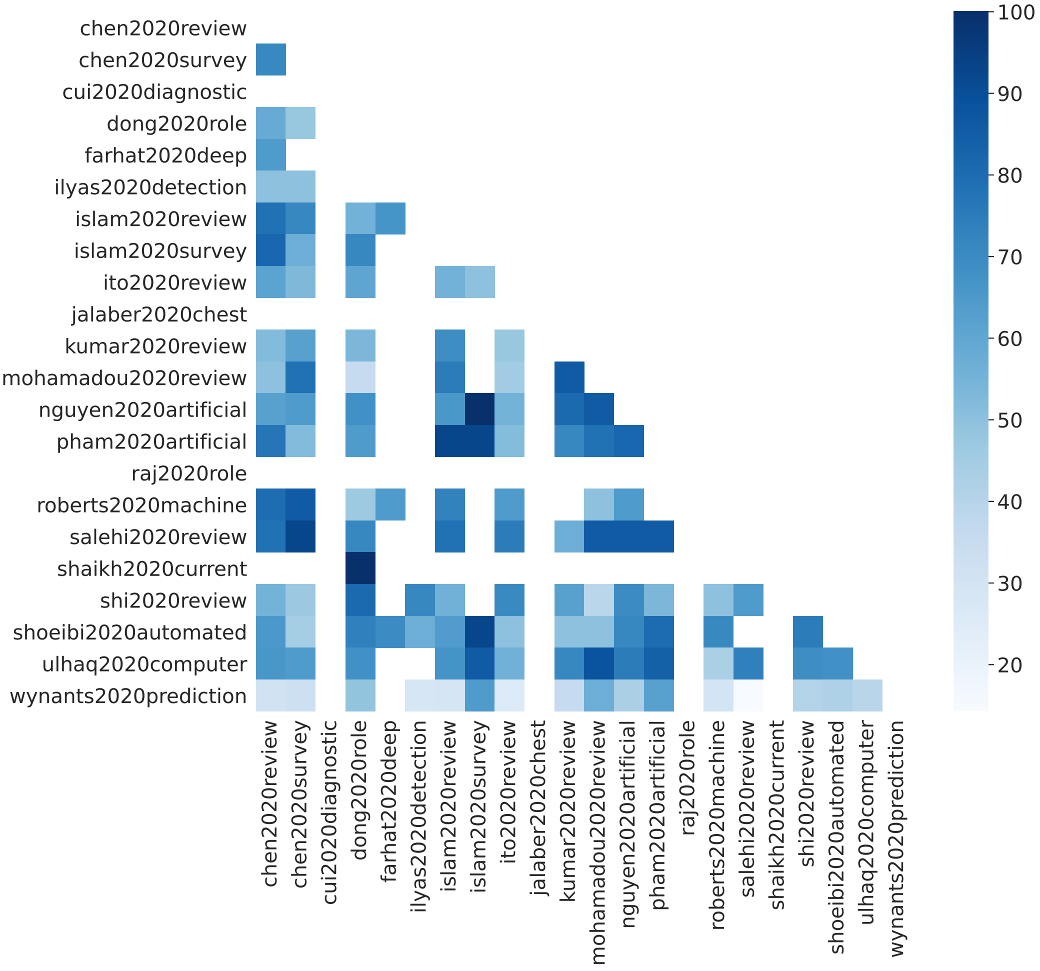


**(a)** Heatmap.

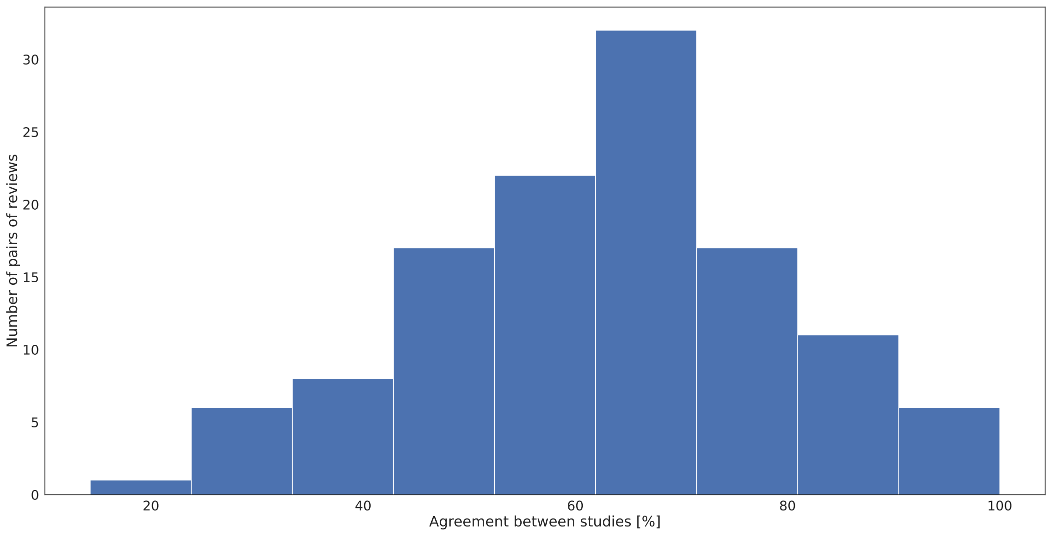


**(b)** Histogram.

**Figure S12.** Level of agreement based on overlapping in extracted data (characteristics only, without text data) between included reviews; analysis A.

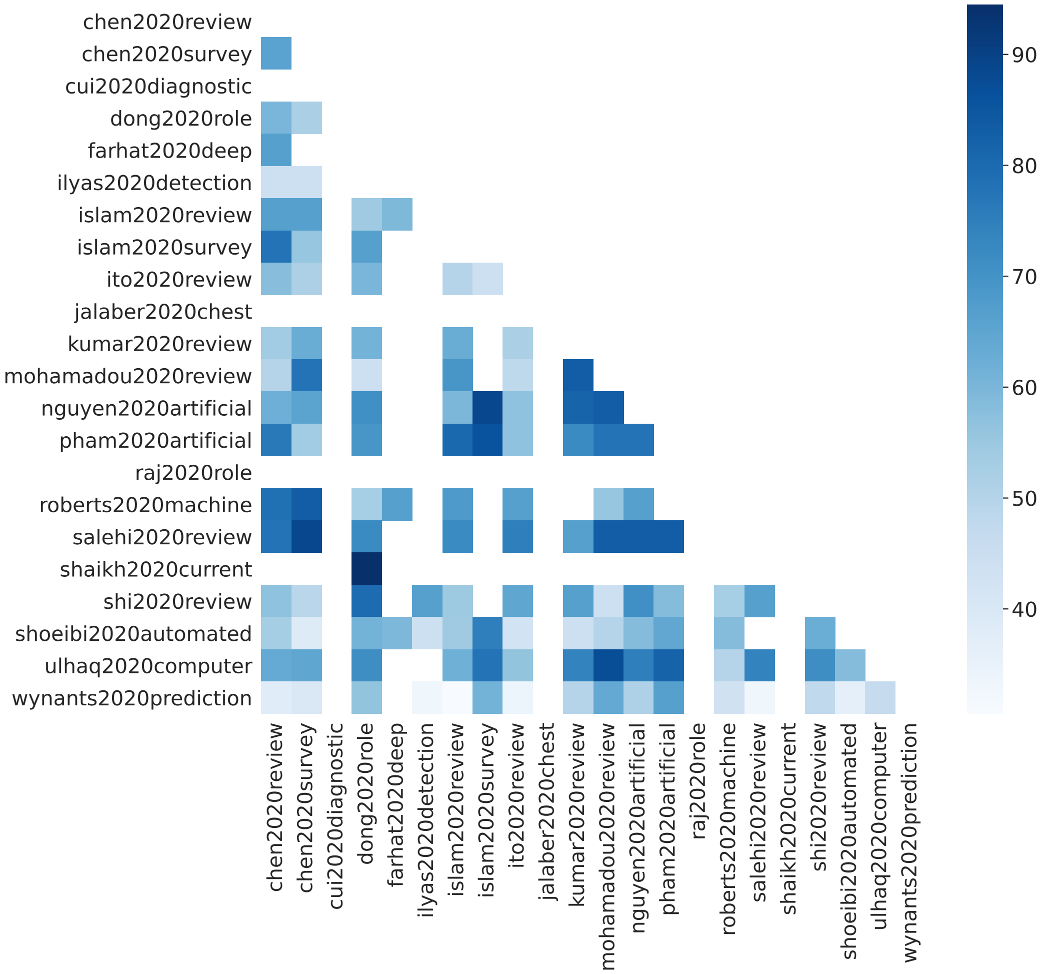


**(a)** Heatmap.

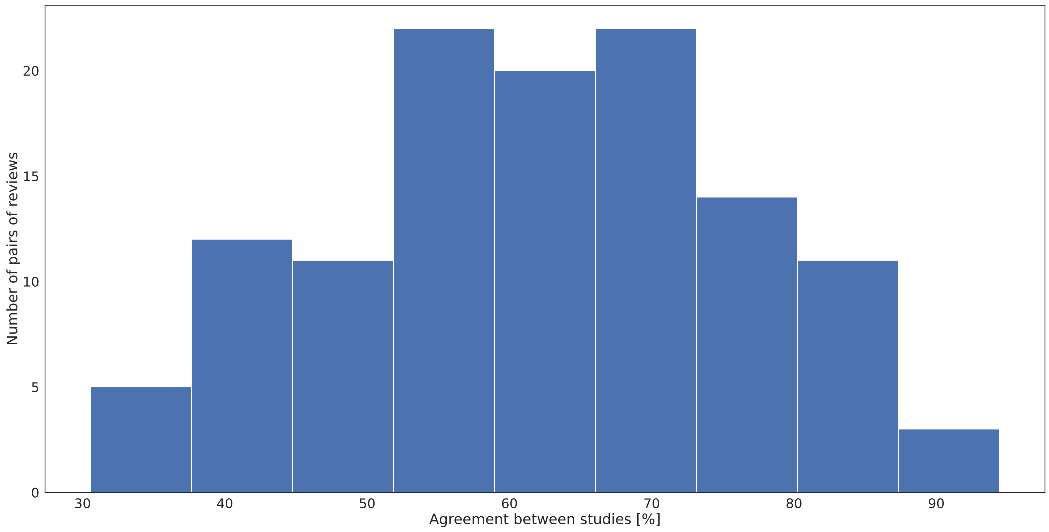


**(b)** Histogram.

**Figure S13.** Level of agreement based on overlapping in extracted data (characteristics only) between included reviews; analysis A.

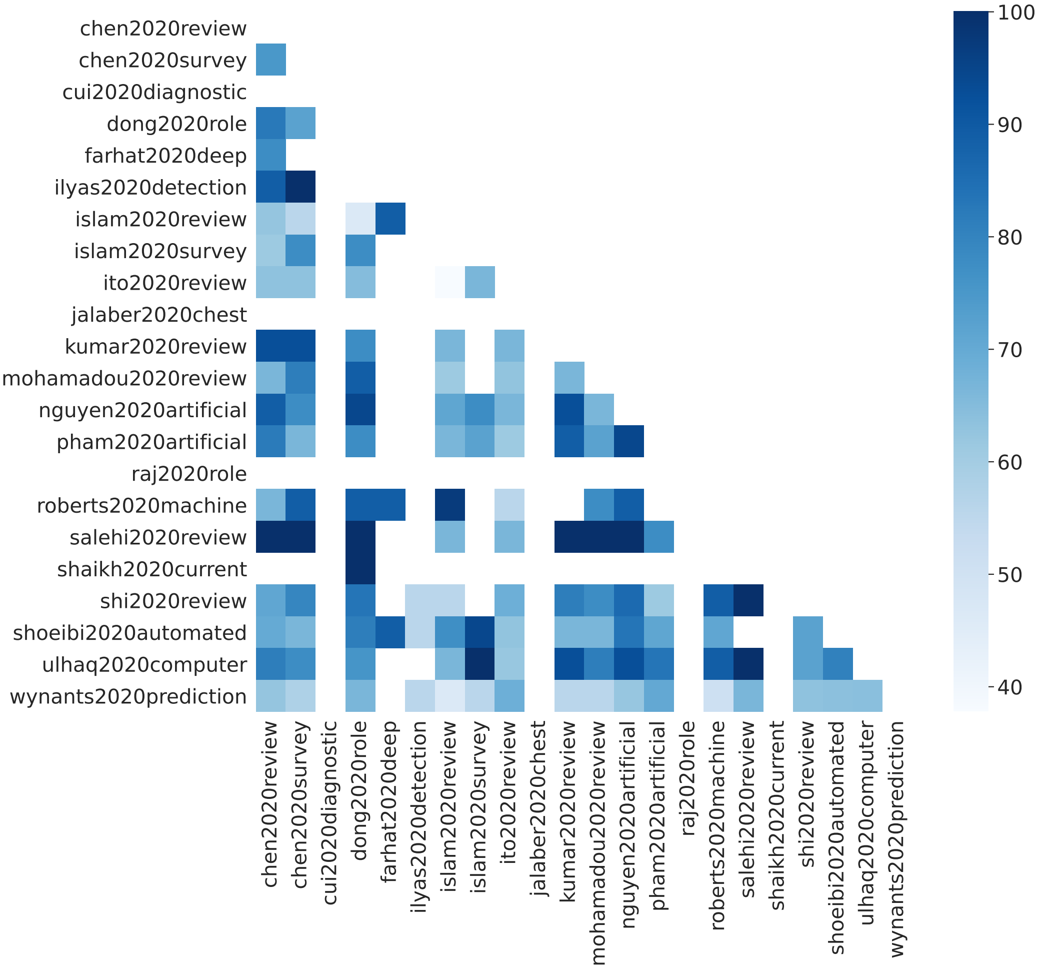


**(a)** Heatmap.

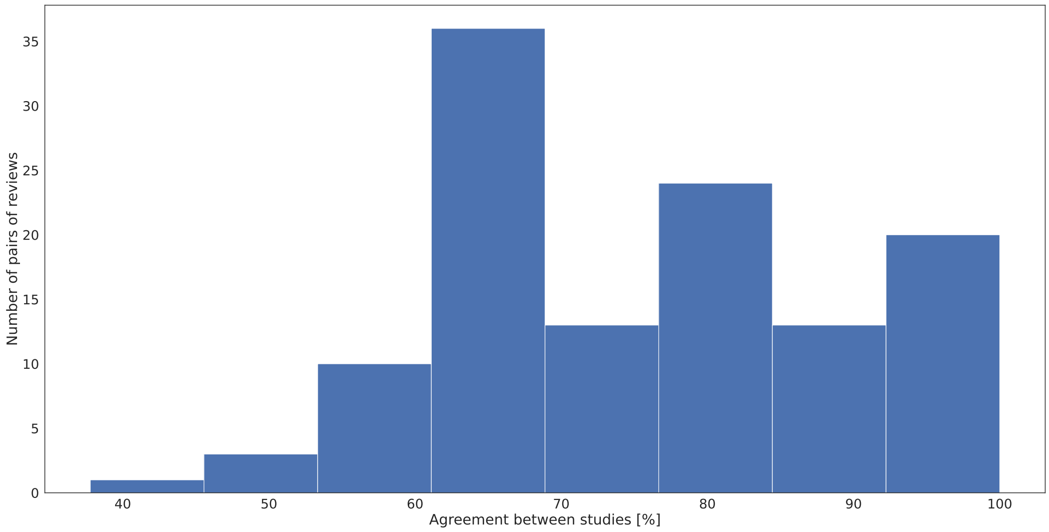


**(b)** Histogram.

**Figure S14.** Level of agreement based on overlapping in extracted data (outcomes only) between included reviews; analysis A.

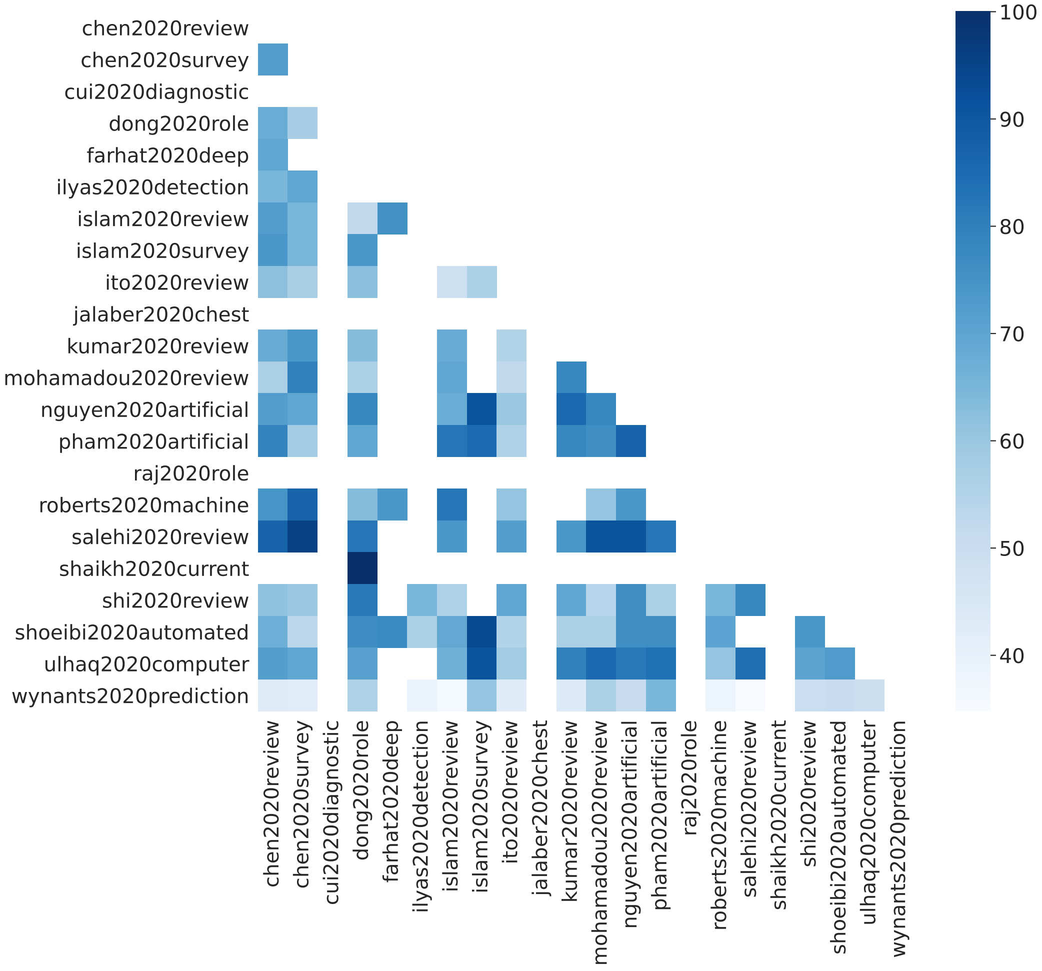


**(a)** Heatmap.

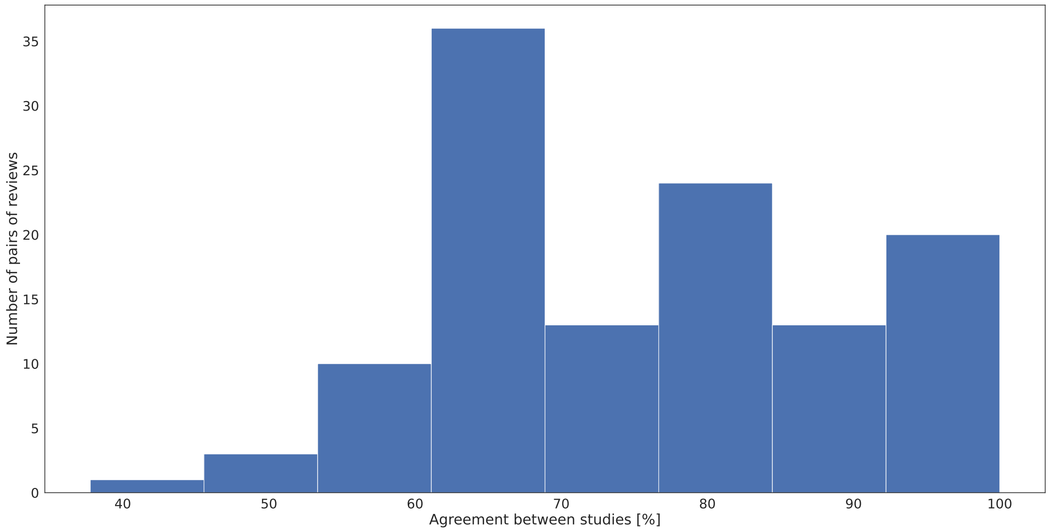


**(b)** Histogram.

**Figure S15.** Level of agreement based on overlapping in extracted data (all variables, without text data) between included reviews; analysis A.

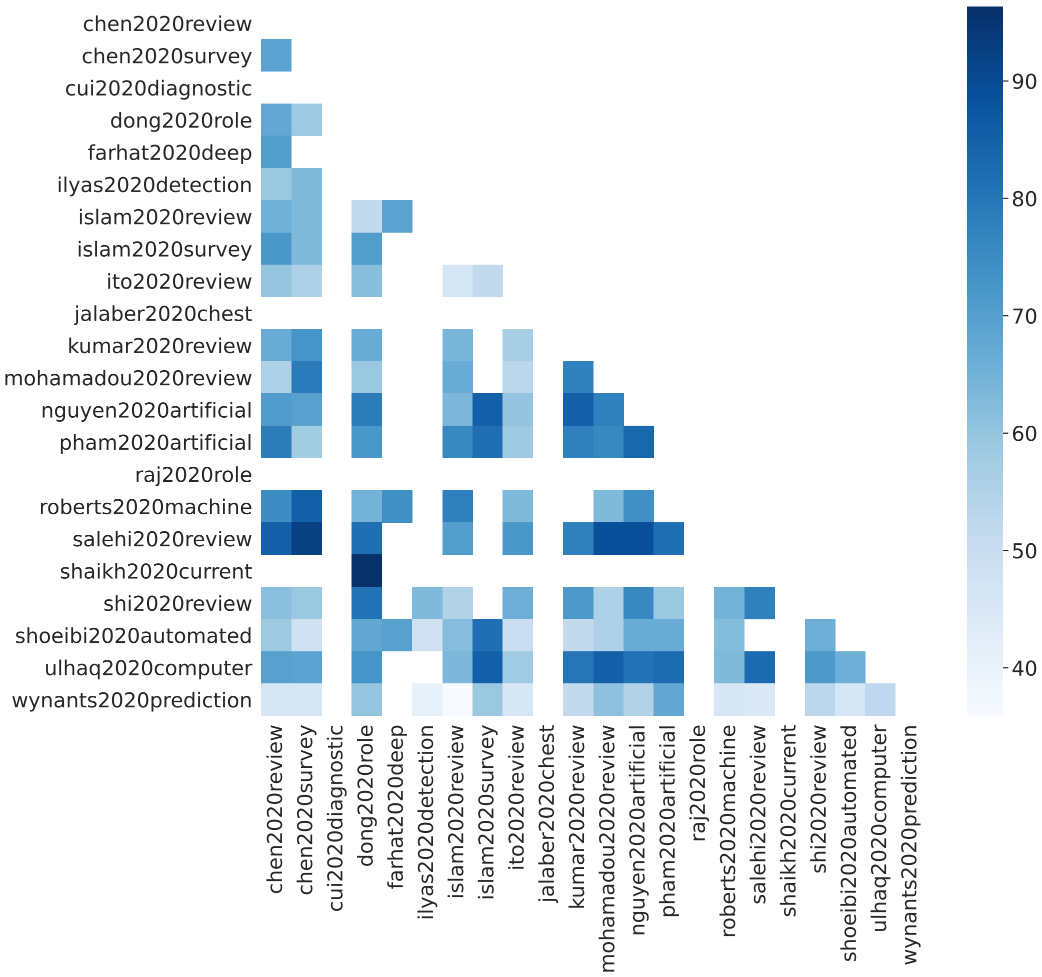


**(a)** Heatmap.

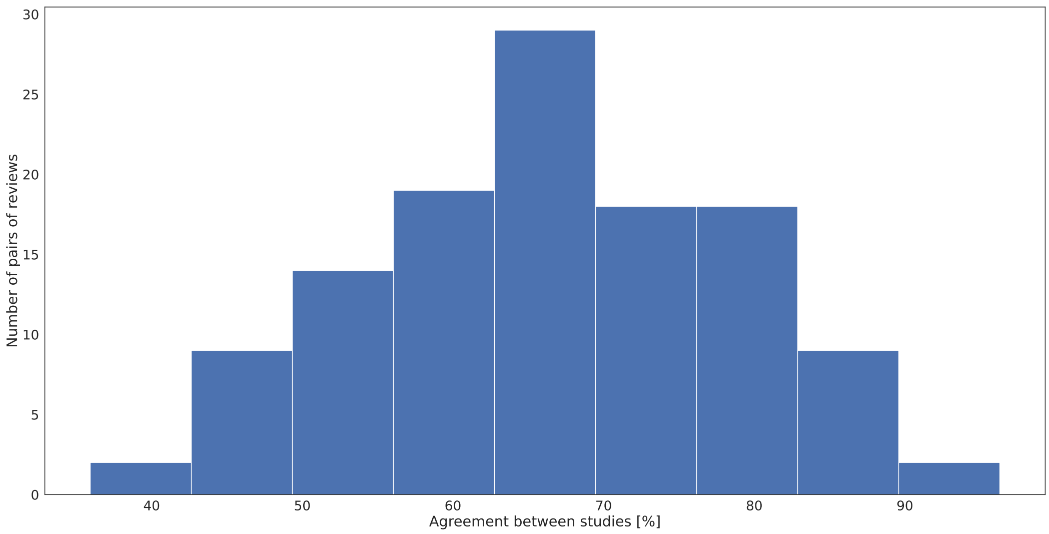


**(b)** Histogram.

**Figure S16.** Level of agreement based on overlapping in extracted data (all variables) between included reviews; analysis A.

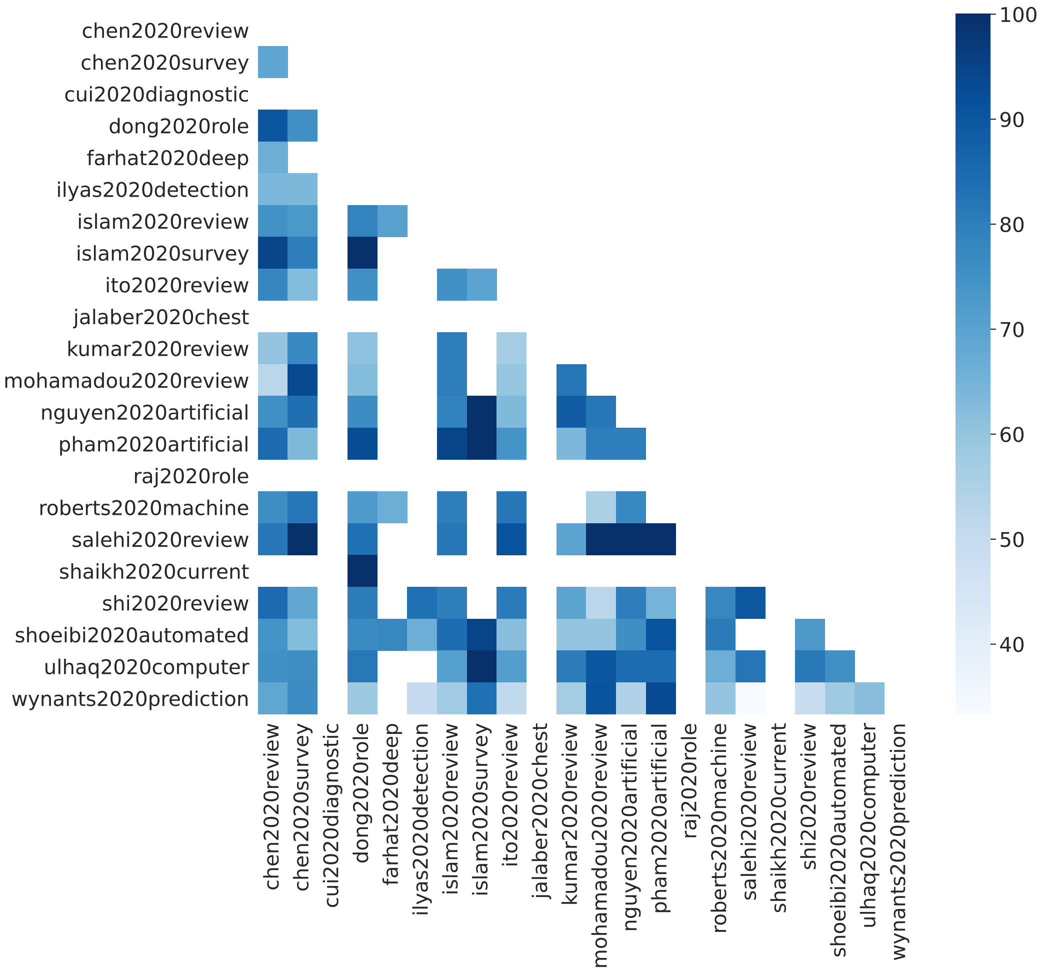


**(a)** Heatmap.

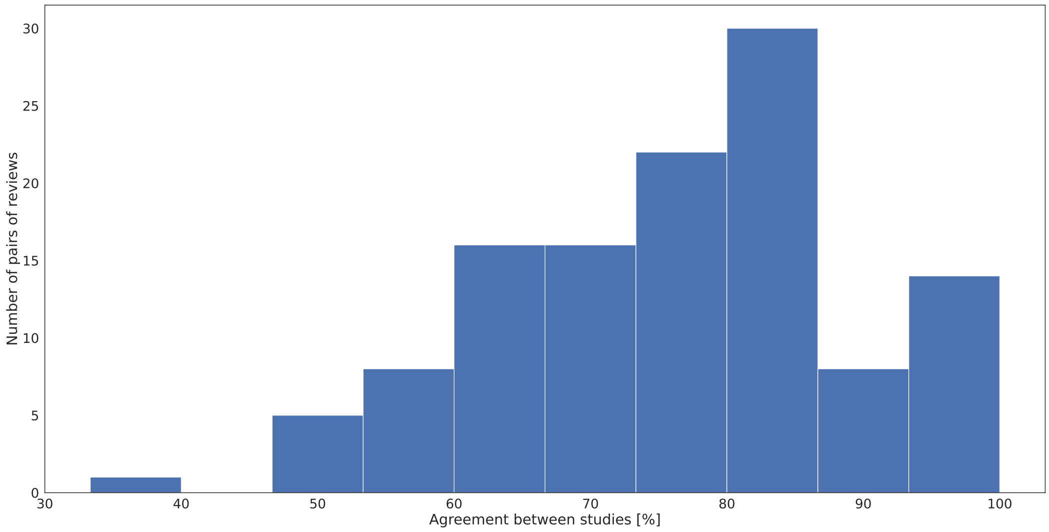


**(b)** Histogram.

**Figure S17.** Level of agreement based on overlapping in extracted data (characteristics only, without text data) between included reviews; analysis B.

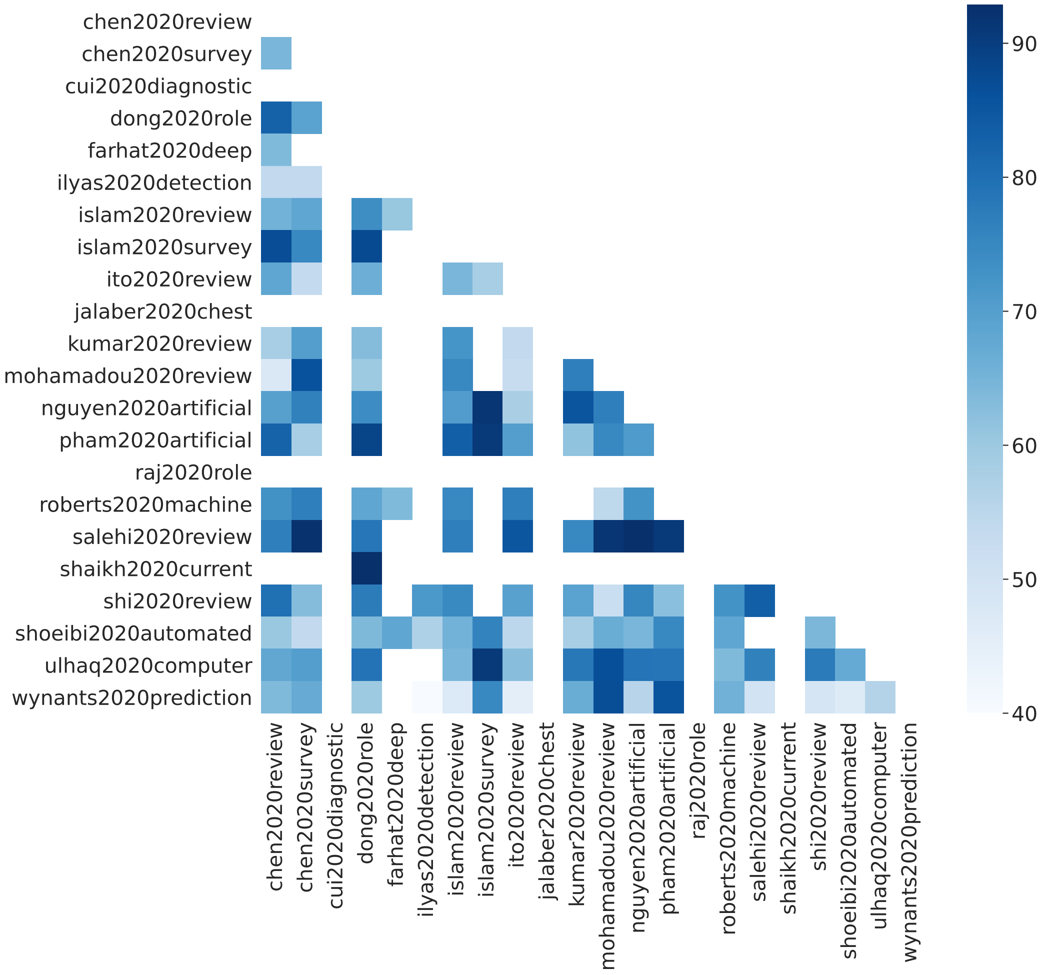


**(a)** Heatmap.

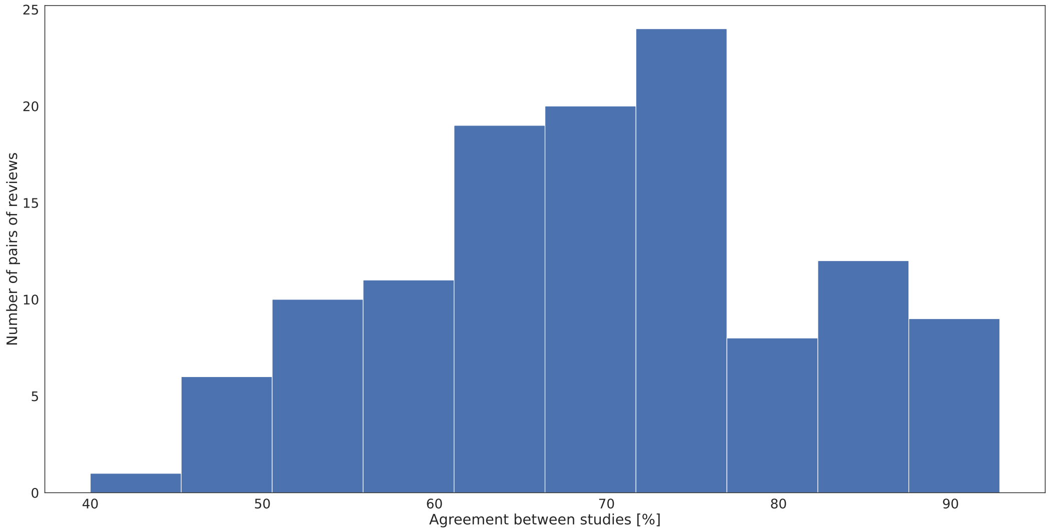


**(b)** Histogram.

**Figure S18.** Level of agreement based on overlapping in extracted data (characteristics only) between included reviews; analysis B.

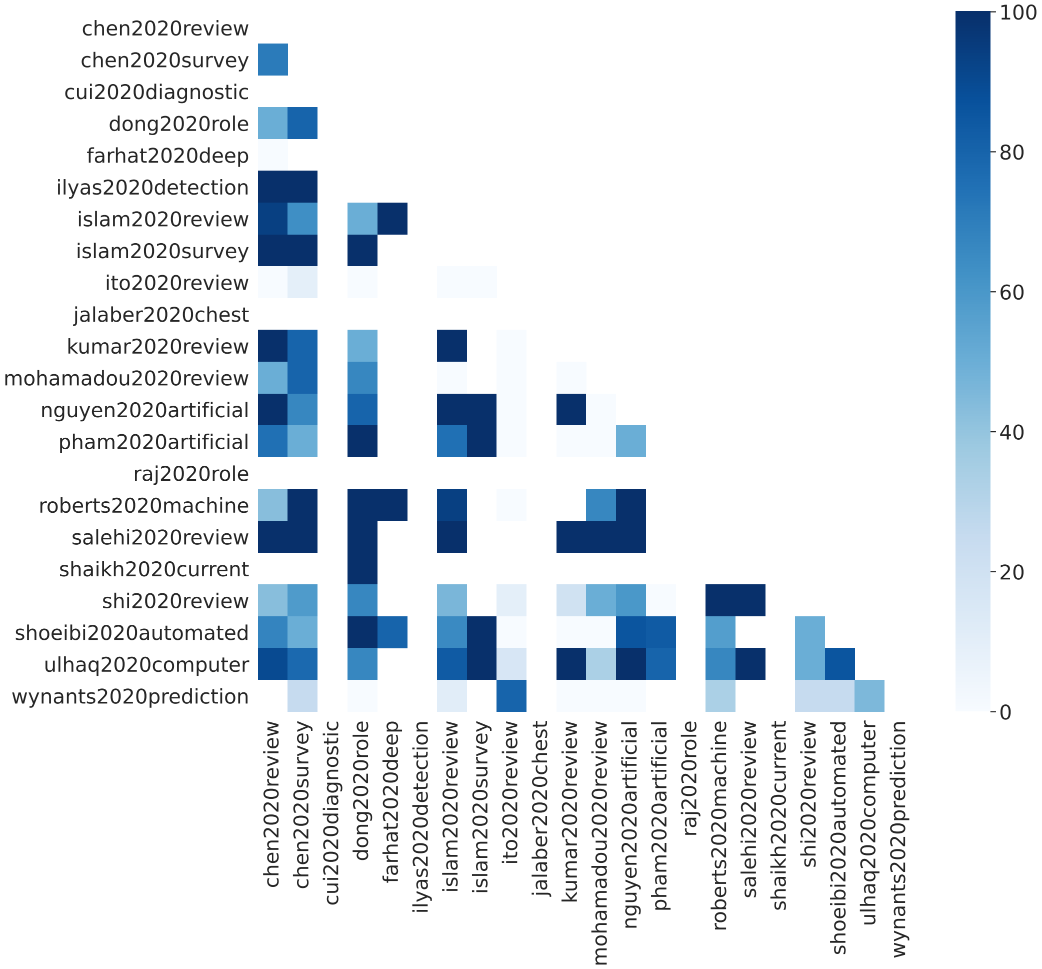


**(a)** Heatmap.

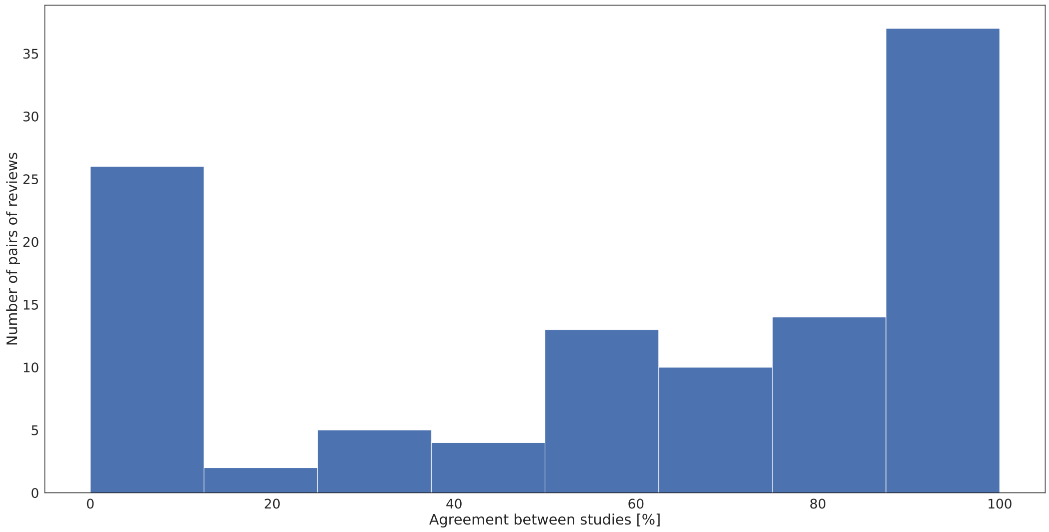


**(b)** Histogram.

**Figure S19.** Level of agreement based on overlapping in extracted data (outcomes only) between included reviews; analysis B.

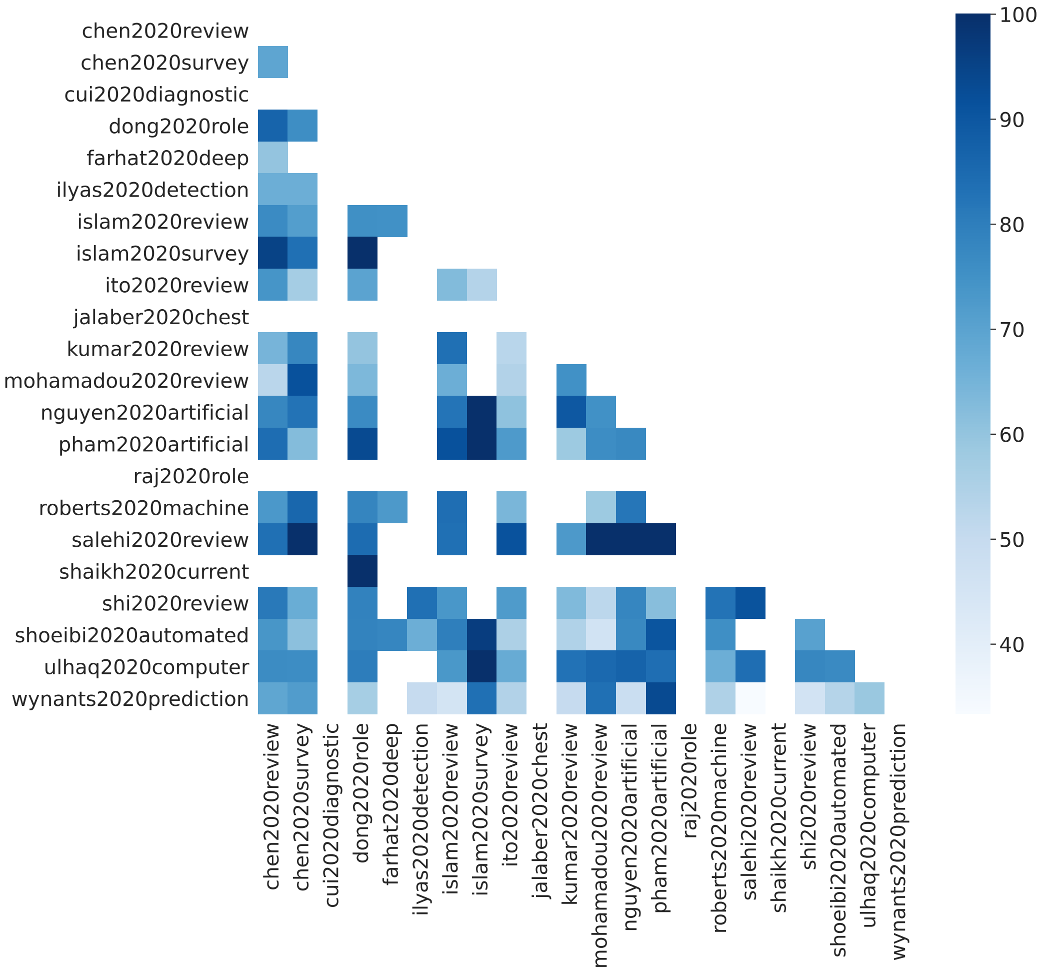


**(a)** Heatmap.

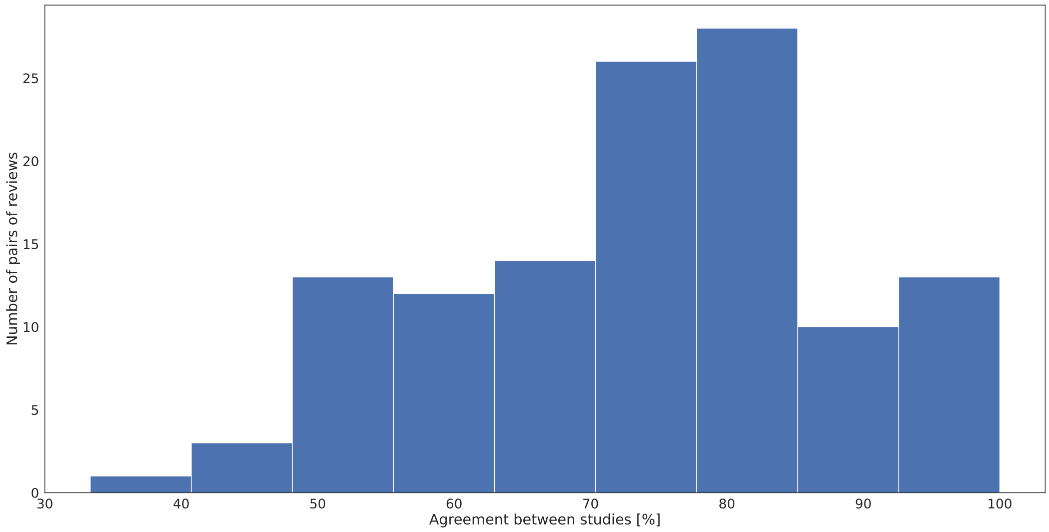


**(b)** Histogram.

**Figure S20.** Level of agreement based on overlapping in extracted data (all variables, without text data) between included reviews; analysis B.

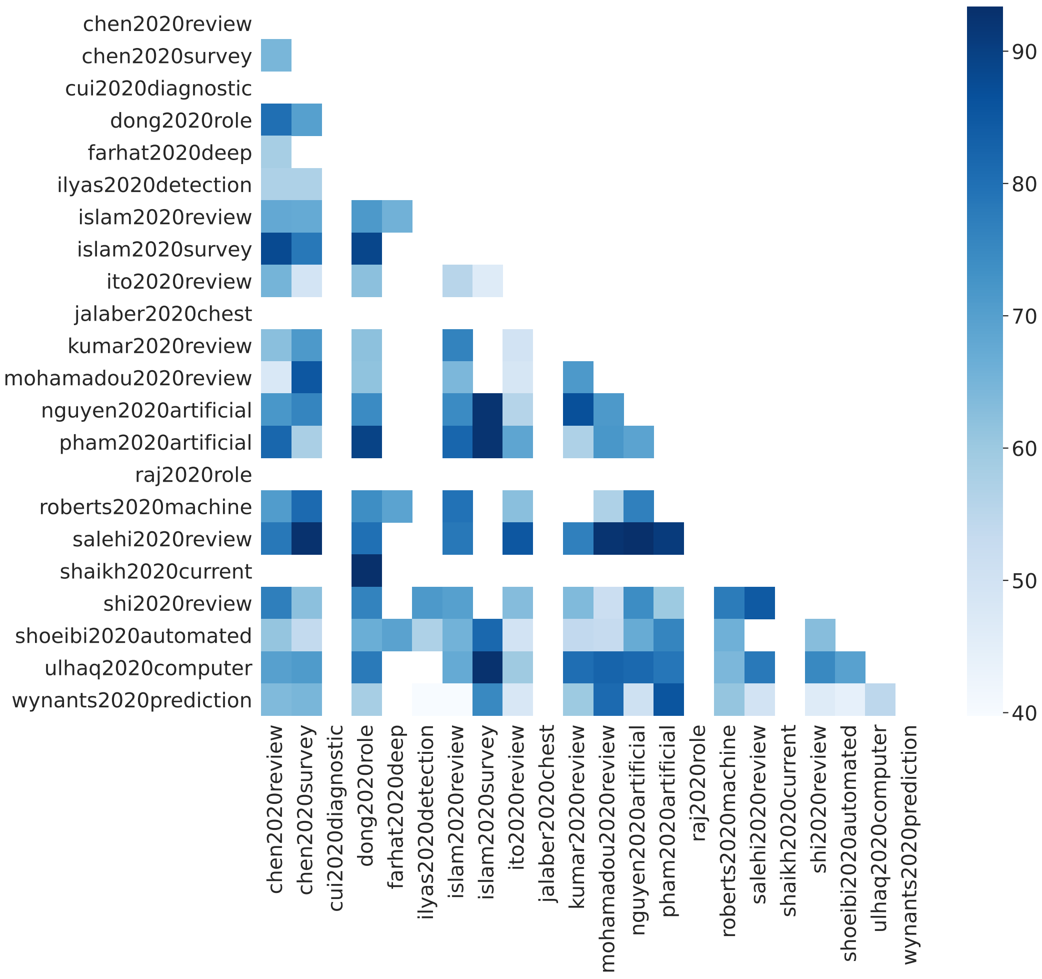


**(a)** Heatmap.

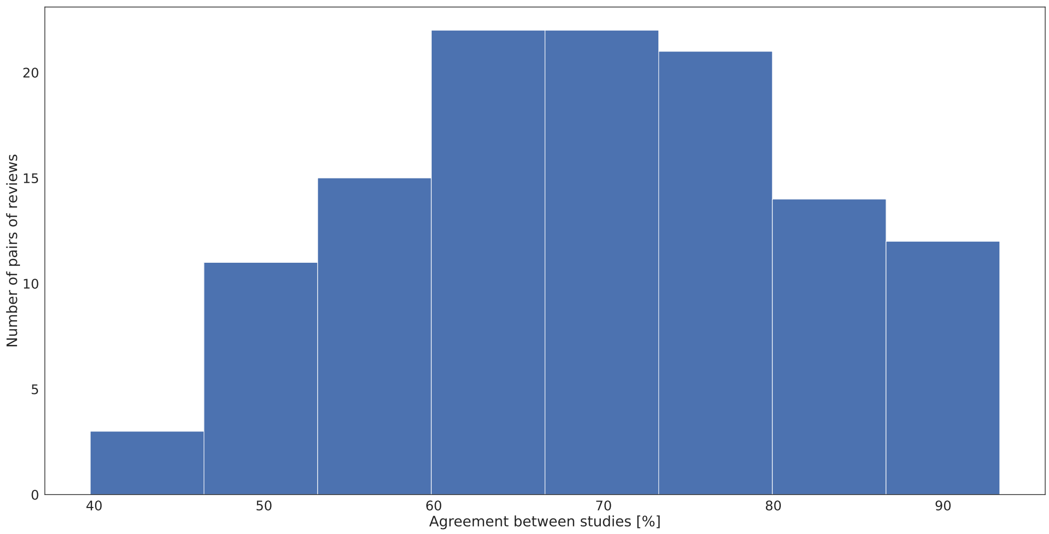


**(b)** Histogram.

**Figure S21.** Level of agreement based on overlapping in extracted data (all variables) between included reviews; analysis B.

**Text S1. Search Strategies**

*MEDLINE*

1. exp Coronavirus Infections/ or exp Coronavirus/ or exp Betacoronavirus/ or ("Coronavirus Infection*" or "Coronavirus" or "Betacoronavirus").ti,ab. or (("corona*" or "corono*") adj1 ("virus*" or "viral*" or "virinae*")).ti,ab. or ("coronavirus*" or "Severe acute respiratory syndrome related coronavirus" or "Severe acute respiratory syndrome coronavirus 2" or "coronovirus*" or "coron?virinae*" or "2019-nCoV" or "2019nCoV" or "2019-CoV" or "nCoV2019" or "nCoV-2019" or "COVID-19" or "COVID19" or "CORVID-19" or "CORVID19" or "WN-CoV" or "WNCoV" or "HCoV-19" or "HCoV19" or "CoV" or "2019 novel*" or "2019 novel coronavirus" or "2019 nCoV" or "Ncov" or "n-cov" or "SARS-CoV-2" or "SARSCoV-2" or "SARSCoV2" or "SARS-CoV2" or "SARSCov19" or "SARS-Cov19" or "SARSCov-19" or "SARS-Cov-19" or "SARSr-cov" or "Ncovor" or "Ncorona*" or "Ncorono*" or "NcovWuhan*" or "NcovHubei*" or "NcovChina*" or "NcovChinese*" or "Wuhan virus*" or "novel CoV" or "CoV 2" or "CoV2" or "betacoron?vir*").ti,ab. or ((("respiratory*" adj2 ("acute*" or "symptom*" or "disease*" or "illness*" or "condition*")) or "sea-food market*" or "seafood market*" or "food market*" or "foodmarket*") adj10 ("Wuhan*" or "Hubei*" or "China*" or "Chinese*" or "Huanan*")).ti,ab. or (("outbreak*" or "wildlife*" or "wild-life" or "pandemic*" or "epidemic*") adj3 ("Wuhan*" or "Hubei*" or "China*" or "Chinese*" or "Huanan*")).ti,ab. or ("anti-flu*" or "anti-influenza*" or "antiflu*" or "antinfluenza*").ti,ab.
2. ("influenza" or "AIDS" or "immunodeficiency virus" or "HIV" or "sexually transmitted disease" or "sexually transmitted infections" or "STD" or "STI").ti,ab.
3. ("recogni*" or "classif*" or "regress*" or "clusteri*" or "discriminat*" or "detect*" or "categori*" or "estimat*").ti,ab.
4. ("Machine Learning" or "DL" or "Deep Learning" or "Representation Learning" or "Transfer Learning" or "AI" or "Artificial intelligen*" or "Computational Intelligen*").ti,ab.
5. ("MLP" or (("multi-layer" or "multi layer") and "perceptron") or "LSTM" or "BLSTM" or "GAN" or "generative adversarial" or "RNN" or "ANN" or "DNN" or "CNN" or "NN" or "Neural Network*" or "SVM" or "SVC" or "support vector*" or "LDA" or "QDA" or "discriminant analysis" or "naive bayes*" or "knn" or "nearest neighb*" or "Decision*" or "Expert*" or (("Logistic" or "Linear") AND "Regress*") or "Random Forest" or "Gradient Boost*" or "AdaBoost" or "XGBoost" or "LightGBM" or "classifier*" or "regressor*").ti,ab.
6. exp diagnostic imaging/ or exp diagnosis computer assisted/ or exp Tomography, Emission Computed/ or exp Tomography, X-ray computed/ or exp echography/ or exp magnetic resonance imaging/ or ("diagnostic imaging" or "computer assisted" or "computer-assisted" or "Tomography" or "Emission Computed" or "Emission-Computed" or "X-ray computed" or "X ray computed" or "X-ray-computed" or "echography" or "magnetic resonance imaging" or "mri" or "magnetic resonance imaging" or "microscop*" or "photograph*" or "holograph*" or "radiograph*" or "spectroscop*" or "stroboscop*" or "subtraction technique*" or "thermograph*" or "tomograph*" or "transilluminat*" or "ultrasonograph*" or "ultrasound" or "imaging" or "scan*" or "X-Ray" or "X Ray" or "CT Scan" or "Computed Tomography" or "CT" or "PET" or "PET-CT" or "positron emission tomograph*" or "MRI" or "fMRI" or "NMRI" or "scintigraph*" or "Doppler echography" or "sonograph*" or "ultraso*" or "doppler" or "magnetic resonance imag*").ti,ab.
7. ("overview*" or "review*" or "survey*").af. or exp review/
8. (1 not 2) and (3 or 4 or 5) and 6 and 7

*EMBASE*

1. 'coronavirus infections'/exp OR 'coronavirus'/exp OR 'betacoronavirus'/exp OR 'coronavirus infection*':ti,ab OR 'coronavirus':ti,ab OR 'betacoronavirus':ti,ab OR ((('corona*' OR 'corono*') NEAR/1 ('virus*' OR 'viral*' OR 'virinae*')):ti,ab) OR 'coronavirus*':ti,ab OR 'severe acute respiratory syndrome related coronavirus':ti,ab OR 'severe acute respiratory syndrome coronavirus 2':ti,ab OR 'coronovirus*':ti,ab OR 'coron?virinae*':ti,ab OR '2019-ncov':ti,ab OR '2019ncov':ti,ab OR '2019-cov':ti,ab OR 'ncov2019':ti,ab OR 'ncov-2019':ti,ab OR 'covid-19':ti,ab OR 'covid19':ti,ab OR 'corvid-19':ti,ab OR 'corvid19':ti,ab OR 'wn-cov':ti,ab OR 'wncov':ti,ab OR 'hcov-19':ti,ab OR 'hcov19':ti,ab OR 'cov':ti,ab OR '2019 novel*':ti,ab OR '2019 novel coronavirus':ti,ab OR '2019 ncov':ti,ab OR 'ncov':ti,ab OR 'n-cov':ti,ab OR 'sars-cov-2':ti,ab OR 'sarscov-2':ti,ab OR 'sarscov2':ti,ab OR 'sars-cov2':ti,ab OR 'sarscov19':ti,ab OR 'sars-cov19':ti,ab OR 'sarscov-19':ti,ab OR 'sars-cov-19':ti,ab OR 'sarsr-cov':ti,ab OR 'ncovor':ti,ab OR 'ncorona*':ti,ab OR 'ncorono*':ti,ab OR 'ncovwuhan*':ti,ab OR 'ncovhubei*':ti,ab OR 'ncovchina*':ti,ab OR 'ncovchinese*':ti,ab OR 'wuhan virus*':ti,ab OR 'novel cov':ti,ab OR 'cov 2':ti,ab OR 'cov2':ti,ab OR 'betacoron?vir*':ti,ab OR ((('outbreak*' OR 'wildlife*' OR 'wild-life' OR 'pandemic*' OR 'epidemic*') NEAR/3 ('wuhan*' OR 'hubei*' OR 'china*' OR 'chinese*' OR 'huanan*')):ti,ab) OR 'anti-flu*':ti,ab OR 'anti-influenza*':ti,ab OR 'antiflu*':ti,ab OR 'antinfluenza*':ti,ab OR

((('respiratory*' OR 'sea-food market*' OR 'seafood market*' OR 'food market*' OR 'foodmarket*') NEAR/10 ('wuhan*' OR 'hubei*' OR 'china*' OR 'chinese*' OR 'huanan*')):ti,ab)

2. 'influenza':ti,ab OR 'aids':ti,ab OR 'immunodeficiency virus':ti,ab OR 'hiv':ti,ab OR 'sexually transmitted disease':ti,ab OR 'sexually transmitted infections':ti,ab OR 'std':ti,ab OR 'sti':ti,ab
3. 'recogni*':ti,ab OR 'classif*':ti,ab OR 'regress*':ti,ab OR 'clusteri*':ti,ab OR 'discriminat*':ti,ab OR 'detect*':ti,ab OR 'categori*':ti,ab OR 'estimat*':ti,ab
4. 'machine learning':ti,ab OR 'dl':ti,ab OR 'deep learning':ti,ab OR 'representation learning':ti,ab OR 'transfer learning':ti,ab OR 'ai':ti,ab OR 'artificial intelligen*':ti,ab OR 'computational intelligen*':ti,ab
5. 'mlp':ti,ab OR (('multi-layer':ti,ab OR 'multi layer':ti,ab) AND 'perceptron':ti,ab) OR 'lstm':ti,ab OR 'blstm':ti,ab OR 'gan':ti,ab OR 'generative adversarial':ti,ab OR 'rnn':ti,ab OR 'ann':ti,ab OR 'dnn':ti,ab OR 'cnn':ti,ab OR 'nn':ti,ab OR 'neural network*':ti,ab OR 'svm':ti,ab OR 'svc':ti,ab OR 'support vector*':ti,ab OR 'lda':ti,ab OR 'qda':ti,ab OR 'discriminant analysis':ti,ab OR 'naive bayes*':ti,ab OR 'knn':ti,ab OR 'nearest neighb*':ti,ab OR 'decision*':ti,ab OR 'expert*':ti,ab OR (('logistic':ti,ab OR 'linear':ti,ab) AND 'regress*':ti,ab) OR 'random forest':ti,ab OR 'gradient boost*':ti,ab OR 'adaboost':ti,ab OR 'xgboost':ti,ab OR 'lightgbm':ti,ab OR 'classifier*':ti,ab OR 'regressor*':ti,ab
6. 'diagnostic imaging'/exp OR 'diagnosis computer assisted'/exp OR 'tomography, emission computed' /exp OR 'tomography, x-ray computed'/exp OR 'echography'/exp OR 'magnetic resonance imaging'/exp OR 'diagnostic imaging':ti,ab OR 'computer assisted':ti,ab OR 'computer-assisted':ti,ab OR 'tomography':ti,ab OR 'emission computed':ti,ab OR 'emission-computed':ti,ab OR 'x-ray computed': ti,ab OR 'x ray computed':ti,ab OR 'x-ray-computed':ti,ab OR 'echography':ti,ab OR 'magnetic resonance imaging':ti,ab OR 'microscop*':ti,ab OR 'photograph*':ti,ab OR 'holograph*':ti,ab OR 'radiograph*':ti,ab OR 'spectroscop*':ti,ab OR 'stroboscop*':ti,ab OR 'subtraction technique*':ti,ab OR 'thermograph*':ti,ab OR 'tomograph*':ti,ab OR 'transilluminat*':ti,ab OR 'ultrasonograph*':ti,ab OR 'ultrasound':ti,ab OR 'imaging':ti,ab OR 'scan*':ti,ab OR 'x-ray':ti,ab OR 'x ray':ti,ab OR 'ct scan':ti,ab OR 'computed tomography':ti,ab OR 'ct':ti,ab OR 'pet':ti,ab OR 'pet-ct':ti,ab OR ('positron':ti,ab AND 'emission':ti,ab AND 'tomograph*':ti,ab) OR 'mri':ti,ab OR 'fmri':ti,ab OR 'nmri':ti,ab OR 'scintigraph*':ti,ab OR ('doppler':ti,ab AND 'echography':ti,ab) OR 'sonograph*':ti,ab OR 'ultraso*': ti,ab OR 'doppler':ti,ab OR ('magnetic':ti,ab AND 'resonance':ti,ab AND 'imag*':ti,ab)
7. overview* OR review* OR survey* OR 'review'/exp
8. (#1 not #2) and (#3 or #4 or #5) and #6 and #7

*Web of Science*

1. TS=('Coronavirus Infection*' or 'Coronavirus' or 'Betacoronavirus') or TS=(('corona*' or 'corono*') NEAR/1 ('virus*' or 'viral*' or 'virinae*')) or TS=('coronavirus*' or 'Severe acute respiratory syndrome related coronavirus' or 'Severe acute respiratory syndrome coronavirus 2' or 'coronovirus*' or 'coron?virinae*' or '2019-nCoV' or '2019nCoV' or '2019-CoV' or 'nCoV2019' or 'nCoV-2019' or 'COVID-19' or 'COVID19' or 'CORVID-19' or 'CORVID19' or 'WN-CoV' or 'WNCoV' or 'HCoV-19' or 'HCoV19' or 'CoV' or '2019 novel*' or '2019 novel coronavirus' or '2019 nCoV' or 'Ncov' or 'n-cov' or 'SARS-CoV-2' or 'SARSCoV-2' or 'SARSCoV2' or 'SARS-CoV2' or 'SARSCov19' or 'SARS-Cov19' or 'SARSCov-19' or 'SARS-Cov-19' or 'SARSr-cov' or 'Ncovor' or 'Ncorona*' or 'Ncorono*' or 'NcovWuhan*' or 'NcovHubei*' or 'NcovChina*' or 'NcovChinese*' or 'Wuhan virus*' or 'novel CoV' or 'CoV 2' or 'CoV2' or 'betacoron?vir*') or TS=((('respiratory*' NEAR/2 ('acute*' or 'symptom*' or 'disease*' or 'illness*' or 'condition*')) or 'sea-food market*' or 'seafood market*' or 'food market*' or 'foodmarket*') NEAR/10 ('Wuhan*' or 'Hubei*' or 'China*' or 'Chinese*' or 'Huanan*')) or TS=(('outbreak*' or 'wildlife*' or 'wild-life' or 'pandemic*' or 'epidemic*') NEAR/3 ('Wuhan*' or 'Hubei*' or 'China*' or 'Chinese*' or 'Huanan*')) or TS=('anti-flu*' or 'anti-influenza*' or 'antiflu*' or 'antinfluenza*')
2. TS=('influenza' or 'AIDS' or 'immunodeficiency virus' or 'HIV' or 'sexually transmitted disease' or 'sexually transmitted infections' or 'STD' or 'STI')
3. TS=('recogni*' or 'classif*' or 'regress*' or 'clusteri*' or 'discriminat*' or 'detect*' or 'categori*' or 'estimat*')
4. TS=('Machine Learning' or 'DL' or 'Deep Learning' or 'Representation Learning' or 'Transfer Learning' or 'AI' or 'Artificial intelligen*' or 'Computational Intelligen*')
5. TS=('MLP' or (('multi-layer' or 'multi layer') and 'perceptron') or 'LSTM' or 'BLSTM' or 'GAN' or 'generative adversarial' or 'RNN' or 'ANN' or 'DNN' or 'CNN' or 'NN' or 'Neural Network*' or 'SVM' or 'SVC' or 'support vector*' or 'LDA' or 'QDA' or 'discriminant analysis' or 'naive bayes*' or 'knn' or 'nearest neighb*' or 'Decision*' or 'Expert*' or (('Logistic' or 'Linear') AND 'Regress*') or 'Random Forest' or 'Gradient Boost*' or 'AdaBoost' or 'XGBoost' or 'LightGBM' or 'classifier*' or 'regressor*')

6. TS=('diagnostic imaging' or 'computer assisted' or 'computer-assisted' or 'Tomography' or 'Emission Computed' or 'Emission-Computed' or 'X-ray computed' or 'X ray computed' or 'X-ray-computed' or 'echography' or 'magnetic resonance imaging' or 'mri' or 'magnetic resonance imaging' or 'microscop*' or 'photograph*' or 'holograph*' or 'radiograph*' or 'spectroscop*' or 'stroboscop*' or 'subtraction technique*' or 'thermograph*' or 'tomograph*' or 'transilluminat*' or 'ultrasonograph*' or 'ultrasound' or 'imaging' or 'scan*' or 'X-Ray' or 'X Ray' or 'CT Scan' or 'Computed Tomography' or 'CT' or 'PET' or 'PET-CT' or 'positron emission tomograph*' or 'MRI' or 'fMRI' or 'NMRI' or 'scintigraph*' or 'Doppler echography' or 'sonograph*' or 'ultraso*' or 'doppler' or 'magnetic resonance imag*')
7. ALL=('overview*' or 'review*' or 'survey*')
8. (#1 not #2) and (#3 or #4 or #5) and #6 and #7

*Scopus*

((TITLE-ABS-KEY ("Coronavirus Infection*" OR "Coronavirus" OR "Betacoronavirus") OR TITLE-ABS-KEY (("corona*" OR "corono*") W/1 ("virus*" OR "viral*" OR "virinae*")) OR TITLE-ABS-KEY ("coronavirus*" OR "Severe acute respiratory syndrome related coronavirus" OR "Severe acute respiratory syndrome coronavirus 2" OR "coronovirus*" OR "coron?virinae*" OR "2019-nCoV" OR "2019nCoV" OR "2019-CoV" OR "nCoV2019" OR "nCoV-2019" OR "COVID-19" OR "COVID19" OR "CORVID-19" OR "CORVID19" OR "WN-CoV" OR "WNCoV" OR "HCoV-19" OR "HCoV19" OR "CoV" OR "2019 novel*" OR "2019 novel coronavirus" OR "2019 nCoV" OR "Ncov" OR "n-cov" OR "SARS-CoV-2" OR "SARSCoV-2" OR "SARSCoV2" OR "SARS-CoV2" OR "SARSCov19" OR "SARS-Cov19" OR "SARSCov-19" OR "SARS-Cov-19" OR "SARSr-cov" OR "Ncovor" OR "Ncorona*" OR "Ncorono*" OR "NcovWuhan*" OR "NcovHubei*" OR "NcovChina*" OR "NcovChinese*" OR "Wuhan virus*" OR "novel CoV" OR "CoV 2" OR "CoV2" OR "betacoron?vir*") OR TITLE-ABS-KEY ((("respiratory*" W/2 ("acute*" OR "symptom*" OR "disease*" OR "illness*" OR "condition*")) OR "sea-food market*" OR "seafood market*" OR "food market*" OR "foodmarket*") W/10 ("Wuhan*" OR "Hubei*" OR "China*" OR "Chinese*" OR "Huanan*")) OR TITLE-ABS-KEY (("outbreak*" OR "wildlife*" OR "wild-life" OR "pandemic*" OR "epidemic*") W/3 ("Wuhan*" OR "Hubei*" OR "China*" OR "Chinese*" OR "Huanan*")) OR TITLE-ABS-KEY ("anti-flu*" OR "anti-influenza*" OR "antiflu*" OR "antinfluenza*")) AND NOT (TITLE-ABS-KEY ("influenza" OR "AIDS" OR "immunodeficiency virus" OR "HIV" OR "sexually transmitted disease" OR "sexually transmitted infections" OR "STD" OR "STI"))) AND ((TITLE-ABS-KEY ("recogni*" OR "classif*" OR "regress*" OR "clusteri*" OR "discriminat*" OR "detect*" OR "categori*" OR "estimat*")) OR (TITLE-ABS-KEY ("Machine Learning" OR "DL" OR "Deep Learning" OR "Representation Learning" OR "Transfer Learning" OR "AI" OR "Artificial intelligen*" OR "Computational Intelligen*")) OR (TITLE-ABS-KEY ("MLP" OR "multi-layer perceptron" OR "multi layer perceptron" OR "LSTM" OR "BLSTM" OR "GAN" OR "generative adversarial" OR "RNN" OR "ANN" OR "DNN" OR "CNN" OR "NN" OR "Neural Network*" OR "SVM" OR "SVC" OR "support vector*" OR "LDA" OR "QDA" OR "discriminant analysis" OR "naive bayes*" OR "knn" OR "nearest neighb*" OR "Decision*" OR "Expert*" OR "Logistic Regress*" OR "Linear Regress*" OR "Random Forest" OR "Gradient Boost*" OR "AdaBoost" OR "XGBoost" OR "LightGBM" OR "classifier*" OR "regressor*"))) AND (TITLE-ABS-KEY ("diagnostic imaging" OR "computer assisted" OR "computer-assisted" OR "Tomography" OR "Emission Computed" OR "Emission-Computed" OR "X-ray computed" OR "X ray computed" OR "X-ray-computed" OR "echography" OR "magnetic resonance imaging" OR "mri" OR "magnetic resonance imaging" OR "microscop*" OR "photograph*" OR "holograph*" OR "radiograph*" OR "spectroscop*" OR "stroboscop*" OR "subtraction technique*" OR "thermograph*" OR "tomograph*" OR "transilluminat*" OR "ultrasonograph*" OR "ultrasound" OR "imaging" OR "scan*" OR "X-Ray" OR "X Ray" OR "CT Scan" OR "Computed Tomography" OR "CT" OR "PET" OR "PET-CT" OR "positron emission tomograph*" OR "MRI" OR "fMRI" OR "NMRI" OR "scintigraph*" OR "Doppler echography" OR "sonograph*" OR "ultraso*" OR "doppler" OR "magnetic resonance imag*")) AND (ALL("overview*" OR "review*" OR "survey*"))

*Cochrane Library*

1. MeSH descriptor: [Coronavirus Infections] explode all trees
2. MeSH descriptor: [Coronavirus] explode all trees
3. MeSH descriptor: [Betacoronavirus] explode all trees
4. "coronavirus*" or "Severe acute respiratory syndrome related coronavirus" or "Severe acute respiratory syndrome coronavirus 2" or "coronovirus*" or "coron?virinae*" or "2019-nCoV" or "2019nCoV" or "2019-CoV" or "nCoV2019" or "nCoV-2019" or "COVID-19" or "COVID19" or "CORVID-19" or "CORVID19" or "WN-CoV" or "WNCoV" or "HCoV-19" or "HCoV19" or "CoV" or "2019 novel*" or "2019 novel coronavirus" or "2019 nCoV" or "Ncov" or "n-cov" or "SARS-CoV-2" or "SARSCoV-2" or "SARSCoV2" or "SARS-CoV2" or "SARSCov19" or "SARS-Cov19" or "SARSCov-19"

or "SARS-Cov-19" or "SARSr-cov" or "Ncovor" or "Ncorona*" or "Ncorono*" or "NcovWuhan*" or "NcovHubei*" or "NcovChina*" or "NcovChinese*" or "Wuhan virus*" or "novel CoV" or "CoV 2" or "CoV2" or "betacoron?vir*"

5. ((("respiratory*" NEAR/2 ("acute*" or "symptom*" or "disease*" or "illness*" or "condition*")) or "sea-food market*" or "seafood market*" or "food market*" or "foodmarket*") NEAR/10 ("Wuhan*" or "Hubei*" or "China*" or "Chinese*" or "Huanan*"))
6. (("outbreak*" or "wildlife*" or "wild-life" or "pandemic*" or "epidemic*") NEAR/3 ("Wuhan*" or "Hubei*" or "China*" or "Chinese*" or "Huanan*"))
7. ("anti-flu*" or "anti-influenza*" or "antiflu*" or "antinfluenza*")
8. #1 OR #2 OR #3 OR #4 OR #5 OR #6 OR #7
9. "influenza" or "AIDS" or "immunodeficiency virus" or "HIV" or "sexually transmitted disease" or "sexually transmitted infections" or "STD" or "STI"
10. "recogni*" or "classif*" or "regress*" or "clusteri*" or "discriminat*" or "detect*" or "categori*" or "estimat*"
11. "Machine Learning" or "DL" or "Deep Learning" or "Representation Learning" or "Transfer Learning" or "AI" or "Artificial intelligen*" or "Computational Intelligen*"
12. "MLP" or (("multi-layer" or "multi layer") and "perceptron") or "LSTM" or "BLSTM" or "GAN" or "generative adversarial" or "RNN" or "ANN" or "DNN" or "CNN" or "NN" or "Neural Network*" or "SVM" or "SVC" or "support vector*" or "LDA" or "QDA" or "discriminant analysis" or "naive bayes*" or "knn" or "nearest neighb*" or "Decision*" or "Expert*" or (("Logistic" or "Linear") AND "Regress*") or "Random Forest" or "Gradient Boost*" or "AdaBoost" or "XGBoost" or "LightGBM" or "classifier*" or "regressor*"
13. MeSH descriptor: [Diagnostic Imaging] explode all trees
14. MeSH descriptor: [Diagnosis, Computer-Assisted] explode all trees
15. MeSH descriptor: [Tomography, X-Ray Computed] explode all trees
16. MeSH descriptor: [Tomography, Emission-Computed] explode all trees
17. MeSH descriptor: [Ultrasonography] explode all trees
18. MeSH descriptor: [Magnetic Resonance Imaging] explode all trees
19. #13 or #14 or #15 or #16 or #17 or #18
20. ("diagnostic imaging" or "computer assisted" or "computer-assisted" or "Tomography" or "Emission Computed" or "Emission-Computed" or "X-ray computed" or "X ray computed" or "X-ray-computed" or "echography" or "magnetic resonance imaging" or "mri" or "magnetic resonance imaging" or "microscop*" or "photograph*" or "holograph*" or "radiograph*" or "spectroscop*" or "stroboscop*" or "subtraction technique*" or "thermograph*" or "tomograph*" or "transilluminat*" or "ultrasonograph*" or "ultrasound" or "imaging" or "scan*" or "X-Ray" or "X Ray" or "CT Scan" or "Computed Tomography" or "CT" or "PET" or "PET-CT" or "positron emission tomograph*" or "MRI" or "fMRI" or "NMRI" or "scintigraph*" or "Doppler echography" or "sonograph*" or "ultraso*" or "doppler" or "magnetic resonance imag*")
21. (("overview*" or "review*" or "survey*"))
22. MeSH descriptor: [Review] explode all trees
23. #21 or #22
24. (#8 not #9) and (#10 or #11 or #12) and (#19 or #20) and #23

*IEEE Xplore*

(("Coronavirus Infection" OR "Coronavirus" OR "Betacoronavirus") OR (("corona" OR "corono") NEAR/1 ("virus" OR "viral" OR "virinae")) OR ("Abstract":"coronavirus" OR "Severe acute respiratory syndrome related coronavirus" OR "Severe acute respiratory syndrome coronavirus 2" OR "coronovirus" OR "coron?virinae" OR "2019-nCoV" OR "2019nCoV" OR "2019-CoV" OR "nCoV2019" OR "nCoV-2019" OR "COVID-19" OR "COVID19" OR "CORVID-19" OR "CORVID19" OR "WN-CoV" OR "WNCoV" OR "HCoV-19" OR "HCoV19" OR "CoV" OR "2019 novel" OR "2019 novel coronavirus" OR "2019 nCoV" OR "Ncov" OR "n-cov" OR "SARS-CoV-2" OR "SARSCoV-2" OR "SARSCoV2" OR "SARS-CoV2" OR "SARSCov19" OR "SARS-Cov19" OR "SARSCov-19" OR "SARS-Cov-19" OR "SARSr-cov" OR "Ncovor" OR "Ncorona" OR "Ncorono" OR "NcovWuhan" OR "NcovHubei" OR "NcovChina" OR "NcovChinese" OR "Wuhan virus" OR "novel CoV" OR "CoV 2" OR "CoV2" OR "betacoron?vir") OR ("Document title":"coronavirus" OR "Severe acute respiratory syndrome related coronavirus" OR "Severe acute respiratory syndrome coronavirus 2" OR "coronovirus" OR "coron?virinae" OR "2019-nCoV" OR "2019nCoV" OR "2019-CoV" OR "nCoV2019" OR "nCoV-2019" OR "COVID-19" OR "COVID19" OR "CORVID-19" OR "CORVID19" OR "WN-CoV" OR "WNCoV" OR "HCoV-19" OR "HCoV19" OR "CoV" OR "2019 novel" OR "2019 novel coronavirus" OR "2019 nCoV" OR "Ncov" OR "n-cov" OR "SARS-CoV-2" OR "SARSCoV-2" OR "SARSCoV2" OR "SARS-CoV2" OR "SARSCov19" OR "SARS-Cov19" OR "SARSCov-19" OR "SARS-Cov-19" OR "SARSr-cov" OR

"Ncovor" OR "Ncorona" OR "Ncorono" OR "NcovWuhan" OR "NcovHubei" OR "NcovChina" OR "NcovChinese" OR "Wuhan virus" OR "novel CoV" OR "CoV 2" OR "CoV2" OR "betacoron?vir") OR ((("respiratory" NEAR/2 ("acute" OR "symptom" OR "disease" OR "illness" OR "condition")) OR "sea-food market" OR "seafood market" OR "food market" OR "foodmarket") NEAR/10 ("Wuhan" OR "Hubei" OR "China" OR "Chinese" OR "Huanan")) OR (("outbreak" OR "wildlife" OR "wild-life" OR "pandemic" OR "epidemic") NEAR/3 ("Wuhan" OR "Hubei" OR "China" OR "Chinese" OR "Huanan")) OR ("anti-flu" OR "anti-influenza" OR "antiflu" OR "antinfluenza")) AND (("overview*" OR "review*" OR "survey*") OR "Mesh_Terms":"review")

*dblp*

1. review|overview|survey
2. covid|corona|corono|ncov|wuhan|sars|betaco|corvid|hcov|ncov|hubei|virus|antin|pandem

*arXiv*

1. AND abstract=covid* OR coronavirus; AND abstract=review* OR overview* OR survey*
2. AND title=covid* OR coronavirus; AND abstract=review* OR overview* OR survey*
3. AND abstract=covid* OR coronavirus; AND title=review* OR overview* OR survey*
4. AND title=covid* OR coronavirus; AND title=review* OR overview* OR survey*

*OSF Preprints*

(("Coronavirus Infection*" OR "Coronavirus" OR "Betacoronavirus" OR"corona*" OR "corono*" OR "coronavirus*" OR "Severe acute respiratory syndrome related coronavirus" OR "Severe acute respiratory syndrome coronavirus 2" OR "coronovirus*" OR "coron?virinae*" OR "2019-nCoV" OR "2019nCoV" OR "2019-CoV" OR "nCoV2019" OR "nCoV-2019" OR "COVID-19" OR "COVID19" OR "CORVID-19" OR "CORVID19" OR "WN-CoV" OR "WNCoV" OR "HCoV-19" OR "HCoV19" OR "CoV" OR "2019 novel*" OR "2019 novel coronavirus" OR "2019 nCoV" OR "Ncov" OR "n-cov" OR "SARS-CoV-2" OR "SARSCoV-2" OR "SARSCoV2" OR "SARS-CoV2" OR "SARSCov19" OR "SARS-Cov19" OR "SARSCov-19" OR "SARS-Cov-19" OR "SARSr-cov" OR "NcovOR " OR "Ncorona*" OR "Ncorono*" OR "NcovWuhan*" OR "NcovHubei*" OR "NcovChina*" OR "NcovChinese*" OR "Wuhan virus*" OR "novel CoV" OR "CoV 2" OR "CoV2" OR "betacoron?vir*" OR "respiratory*" OR "sea-food market*" OR "seafood market*" OR "food market*" OR "foodmarket*" OR "outbreak*" OR "wildlife*" OR "wild-life" OR "pandemic*" OR "epidemic*" OR "anti-flu*" OR "anti-influenza*" OR "antiflu*" OR "antinfluenza*") NOT ("influenza" OR "AIDS" OR "immunodeficiency virus" OR "HIV" OR "sexually transmitted disease" OR "sexually transmitted infections" OR "STD" OR "STI")) AND ("review*" OR "overview*" OR "survey*") AND ("diagnostic imaging" OR "computer assisted" OR "computer-assisted" OR "Tomography" OR "Emission Computed" OR "Emission-Computed" OR "X-ray computed" OR "X ray computed" OR "X-ray-computed" OR "echography" OR "magnetic resonance imaging" OR "mri" OR "magnetic resonance imaging" OR "microscop*" OR "photograph*" OR "holograph*" OR "radiograph*" OR "spectroscop*" OR "stroboscop*" OR "subtraction technique*" OR "thermograph*" OR "tomograph*" OR "transilluminat*" OR "ultrasonograph*" OR "ultrasound" OR "imaging" OR "scan*" OR "X-Ray" OR "X Ray" OR "CT Scan" OR "Computed Tomography" OR "CT" OR "PET" OR "PET-CT" OR "positron emission tomograph*" OR "MRI" OR "fMRI" OR "NMRI" OR "scintigraph*" OR "Doppler echography" OR "sonograph*" OR "ultraso*" OR "doppler" OR "magnetic resonance imag*") AND ("recogni*" OR "classif*" OR "regress*" OR "clusteri*" OR "discriminat*" OR "detect*" OR "categori*" OR "estimat*" OR "Machine Learning" OR "DL" OR "Deep Learning" OR "Representation Learning" OR "Transfer Learning" OR "AI" OR "Artificial intelligen*" OR "Computational Intelligen*" OR "MLP" OR "multi-layer perceptron" OR "multi layer perceptron" OR "LSTM" OR "BLSTM" OR "GAN" OR "generative adversarial" OR "RNN" OR "ANN" OR "DNN" OR "CNN" OR "NN" OR "Neural Network*" OR "SVM" OR "SVC" OR "support vector*" OR "LDA" OR "QDA" OR "discriminant analysis" OR "naive bayes*" OR "knn" OR "nearest neighb*" OR "Decision*" OR "Expert*" OR "Logistic Regress*" OR "Linear Regress*" OR "Random Forest" OR "Gradient Boost*" OR "AdaBoost" OR "XGBoost" OR "LightGBM" OR "classifier*" OR "regressor*")

# PRISMA 2020 Checklist

| Section and Topic | Item # | Checklist item | Location where item is reported |
|---|---|---|---|
| **TITLE** | | | |
| Title | 1 | Identify the report as a systematic review. | p. 1 |
| **ABSTRACT** | | | |
| Abstract | 2 | See the PRISMA 2020 for Abstracts checklist. | p. 1 |
| **INTRODUCTION** | | | |
| Rationale | 3 | Describe the rationale for the review in the context of existing knowledge. | pp. 1, 2 |
| Objectives | 4 | Provide an explicit statement of the objective(s) or question(s) the review addresses. | pp. 1, 2 |
| **METHODS** | | | |
| Eligibility criteria | 5 | Specify the inclusion and exclusion criteria for the review and how studies were grouped for the syntheses. | p. 3 |
| Information sources | 6 | Specify all databases, registers, websites, organisations, reference lists and other sources searched or consulted to identify studies. Specify the date when each source was last searched or consulted. | p. 3 |
| Search strategy | 7 | Present the full search strategies for all databases, registers and websites, including any filters and limits used. | S16 |
| Selection process | 8 | Specify the methods used to decide whether a study met the inclusion criteria of the review, including how many reviewers screened each record and each report retrieved, whether they worked independently, and if applicable, details of automation tools used in the process. | p. 3 |
| Data collection process | 9 | Specify the methods used to collect data from reports, including how many reviewers collected data from each report, whether they worked independently, any processes for obtaining or confirming data from study investigators, and if applicable, details of automation tools used in the process. | p. 3 |
| Data items | 10a | List and define all outcomes for which data were sought. Specify whether all results that were compatible with each outcome domain in each study were sought (e.g. for all measures, time points, analyses), and if not, the methods used to decide which results to collect. | p. 3 |
| | 10b | List and define all other variables for which data were sought (e.g. participant and intervention characteristics, funding sources). Describe any assumptions made about any missing or unclear information. | p. 3 |
| Study risk of bias assessment | 11 | Specify the methods used to assess risk of bias in the included studies, including details of the tool(s) used, how many reviewers assessed each study and whether they worked independently, and if applicable, details of automation tools used in the process. | pp. 3, 4 |
| Effect measures | 12 | Specify for each outcome the effect measure(s) (e.g. risk ratio, mean difference) used in the synthesis or presentation of results. | p. 3 |
| Synthesis methods | 13a | Describe the processes used to decide which studies were eligible for each synthesis (e.g. tabulating the study intervention characteristics and comparing against the planned groups for each synthesis (item #5)). | pp. 3, 4, 5 |
| | 13b | Describe any methods required to prepare the data for presentation or synthesis, such as handling of missing summary statistics, or data conversions. | pp. 3, 4, 5 |
| | 13c | Describe any methods used to tabulate or visually display results of individual studies and syntheses. | pp. 3, 4, 5 |
| | 13d | Describe any methods used to synthesize results and provide a rationale for the choice(s). If meta-analysis was performed, describe the model(s), method(s) to identify the presence and extent of statistical heterogeneity, and software package(s) used. | pp. 4, 5 |
| | 13e | Describe any methods used to explore possible causes of heterogeneity among study results (e.g. subgroup analysis, meta-regression). | pp. 4, 5 |
| | 13f | Describe any sensitivity analyses conducted to assess robustness of the synthesized results. | pp. 4, 5 |
| Reporting bias assessment | 14 | Describe any methods used to assess risk of bias due to missing results in a synthesis (arising from reporting biases). | p. 4 |
| Certainty assessment | 15 | Describe any methods used to assess certainty (or confidence) in the body of evidence for an outcome. | NA |

# PRISMA 2020 Checklist

| Section and Topic | Item # | Checklist item | Location where item is reported |
|---|---|---|---|
| **RESULTS** | | | |
| Study selection | 16a | Describe the results of the search and selection process, from the number of records identified in the search to the number of studies included in the review, ideally using a flow diagram. | p. 5, S1 |
| | 16b | Cite studies that might appear to meet the inclusion criteria, but which were excluded, and explain why they were excluded. | S3 |
| Study characteristics | 17 | Cite each included study and present its characteristics. | p. 7, S2, S4 |
| Risk of bias in studies | 18 | Present assessments of risk of bias for each included study. | p. 6, S9, S10 |
| Results of individual studies | 19 | For all outcomes, present, for each study: (a) summary statistics for each group (where appropriate) and (b) an effect estimate and its precision (e.g. confidence/credible interval), ideally using structured tables or plots. | pp. 5, 6, 7, 8 |
| Results of syntheses | 20a | For each synthesis, briefly summarise the characteristics and risk of bias among contributing studies. | pp. 5, 6, 7, 8 |
| | 20b | Present results of all statistical syntheses conducted. If meta-analysis was done, present for each the summary estimate and its precision (e.g. confidence/credible interval) and measures of statistical heterogeneity. If comparing groups, describe the direction of the effect. | NA |
| | 20c | Present results of all investigations of possible causes of heterogeneity among study results. | NA |
| | 20d | Present results of all sensitivity analyses conducted to assess the robustness of the synthesized results. | NA |
| Reporting biases | 21 | Present assessments of risk of bias due to missing results (arising from reporting biases) for each synthesis assessed. | NA |
| Certainty of evidence | 22 | Present assessments of certainty (or confidence) in the body of evidence for each outcome assessed. | NA |
| **DISCUSSION** | | | |
| Discussion | 23a | Provide a general interpretation of the results in the context of other evidence. | pp. 8, 9, 10 |
| | 23b | Discuss any limitations of the evidence included in the review. | p. 10 |
| | 23c | Discuss any limitations of the review processes used. | p. 10 |
| | 23d | Discuss implications of the results for practice, policy, and future research. | pp. 9, 10 |
| **OTHER INFORMATION** | | | |
| Registration and protocol | 24a | Provide registration information for the review, including register name and registration number, or state that the review was not registered. | p. 3 |
| | 24b | Indicate where the review protocol can be accessed, or state that a protocol was not prepared. | p. 3 |
| | 24c | Describe and explain any amendments to information provided at registration or in the protocol. | NA |
| Support | 25 | Describe sources of financial or non-financial support for the review, and the role of the funders or sponsors in the review. | p. 11 |
| Competing interests | 26 | Declare any competing interests of review authors. | p. 11 |
| Availability of data, code and other materials | 27 | Report which of the following are publicly available and where they can be found: template data collection forms; data extracted from included studies; data used for all analyses; analytic code; any other materials used in the review. | p. 11 |

From: [56]
